# APPARILLO: a fully operational and autonomous staring system for LEO debris detection

**DOI:** 10.1007/s12567-021-00380-6

**Published:** 2021-07-05

**Authors:** Paul Wagner, Tim Clausen

**Affiliations:** grid.7551.6 0000 0000 8983 7915 DLR, Institute of Technical Physics, Pfaffenwaldring 38-40, 70569 Stuttgart, Germany

**Keywords:** Passive optical staring, Orbital debris, Space surveillance, Space situational awareness, Low earth orbit, Initial space debris detection

## Abstract

For safe operation of active space crafts, the space debris population needs to be continuously scanned, to avoid collisions of active satellites with space debris. Especially the low Earth orbit (LEO) shows higher risks of collisions due to the highest density of orbital debris. Laser ranging stations can deliver highly accurate distance measurements of debris objects allowing precise orbit determination and more effective collision avoidance. However, a laser ranging station needs accurate a priori orbit information to track an orbital object. To detect and track unknown orbital objects in LEO, here, a passive optical staring system is developed for autonomous 24/7 operation. The system is weather-sealed and does not require any service to perform observations. To detect objects, a wide-angle imaging system with 10° field of view equipped with an astronomical CCD camera was designed and set up to continuously observe the sky for LEO objects. The system can monitor and process several passing objects simultaneously without limitations. It automatically starts an observation, processes the images and saves the 2D angular measurements of each object as equatorial coordinates in the TDM standard. This allows subsequent initial orbit determination and handover to a laser tracking system. During campaigns at twilight the system detected up to 36 objects per hour, with high detection efficiencies of LEO objects larger than 1 m^3^. It is shown that objects as small as 0.1 m^3^ can be detected and that the estimated precision of the measurements is about 0.05° or 7 × the pixel scale.

## Introduction

The number of space debris objects is increasing constantly, putting active satellites into a higher risk of collisions. Even small debris particles with a size of 1 cm can cause major damage to a satellite, and this cascading effect causes an exponential growth to the debris population. To avoid collisions between active satellites and debris, the orbital debris population needs to be constantly scanned and cataloged. To keep orbital objects cataloged their position needs to be measured frequently with high precision, which is required for precise orbit determination. Using the resulting orbit predictions, the risk of a collision between a satellite and a debris object can be calculated and collision avoidance maneuvers can be performed subsequently. Of special interest is the LEO as it shows the highest density of debris fragments. For residential space objects (RSO) in LEO, only tracking radar or laser ranging sensors are capable of delivering good enough (radar) or highly accurate (laser ranging) data for predictions. Current prediction uncertainties of debris RSO in LEO are based on radar measurements and require a large safety margin, resulting in 10,000:1 false alert rate [1]. Laser ranging measurements, on the other hand, have an order of magnitude better precision of the distance measurement. This allows much better orbit prediction [2] hence allowing more effective collision avoidance maneuvers between an active satellite and orbital debris [1].

Due to the small field of view (FOV) of a laser tracking system and small laser beam divergence, the RSO needs to be tracked continuously with high accuracy. This requires a priori orbit information of the RSO, which needs to be obtained by a separate sensor network. Currently, only radar systems in staring mode can fulfill this task of initial detection of unknown RSO in LEO. Their downside is their high hardware and operating cost. For this reason, we developed a passive optical staring system to detect and measure unknown orbital objects in LEO for subsequent laser tracking.

We already reported in our first results [3], that such a system is detecting 25% of objects which could not be correlated using the publicly available TLE catalog [4]. The detection efficiency of object with a radar-cross-section (RCS) between 1 and 2 m^2^ was already 50%, and almost 100% for objects with an RCS larger than 2.25 m^2^ [5]. We also demonstrated an instant handover to our tracking telescope (UFO) and redetected objects, like a rocket body, within the 0.27° large field-of-view (FOV) of the tracking camera, without any a priori information [5].

While former activities required manual observation and processing, here we will present a fully operational passive optical surveillance system called APPARILLO (Autonomous Passive Optical Staring Of LEO Flying Objects), which is operational to contribute in a space surveillance network. It is built for 24/7 autonomous operation to detect orbital objects in LEO and export their measured tracklets in the tracking data message (TDM) format [6].

The system is the foundation of future Stare and Chase handover, where an initial orbit determination (IOD) is calculated instantly from the measurements taken by the staring sensor. This orbit prediction will be sent to a tracking telescope which can perform subsequent tracking and laser ranging. The ranging data allow precise orbit determination and cataloging of a newly detected LEO RSO. This concept was previously published [7] and protected under the utility patent DE 20 2017 101 831 U1 in Germany [8].

**Fig. 1:**
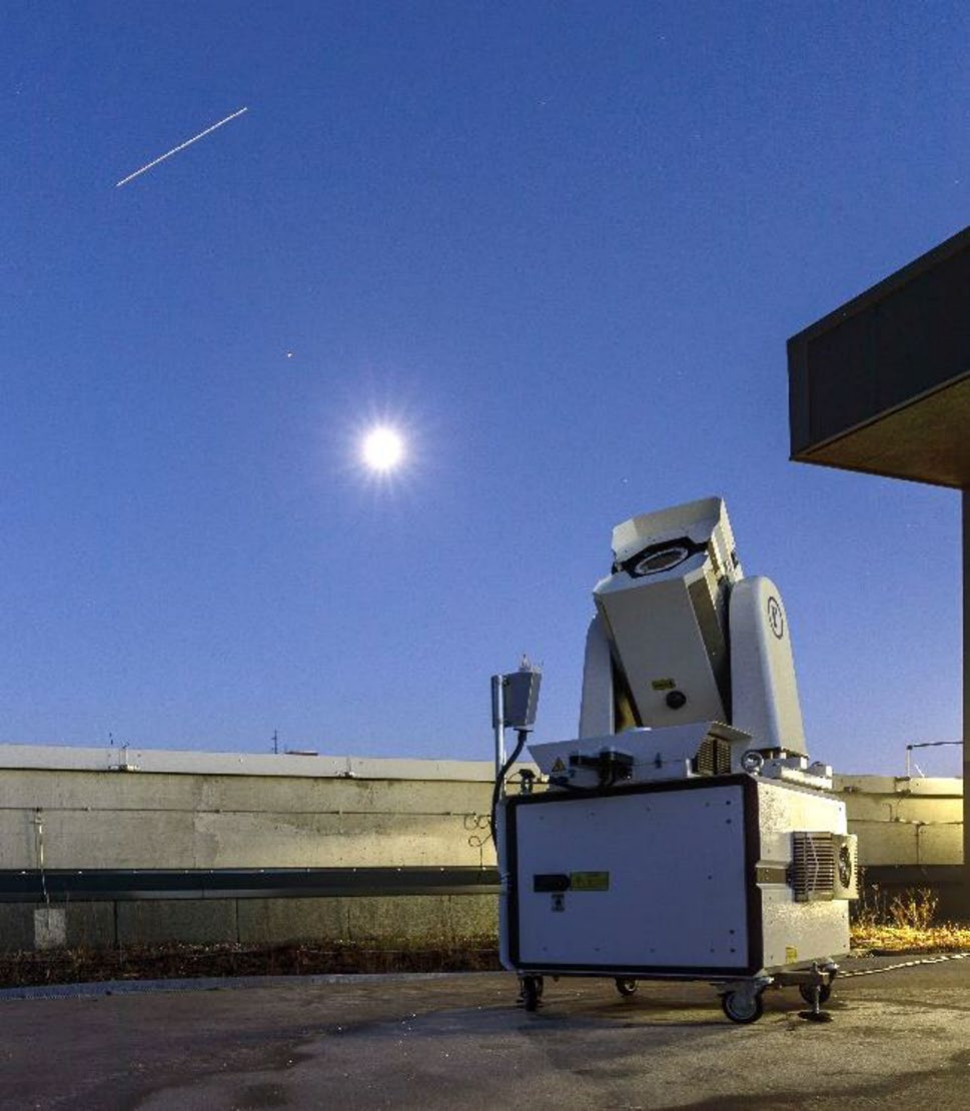
APPARILLO staring system (bottom right) during observation in December 2020. The system has a movable sensor head and a cabinet. In the top of the image is the ISS visible as a streak. The bright object in the center is the full moon and to the top left Mars is visible. The system is located on the top of the DLR office building in Stuttgart, Germany. Credit: Paul Wagner

The current system is a subsequent improvement to ensure autonomous operation, including weather sealing, automatic data recording, and processing. The photograph in Fig. 1 below shows the current system during observation campaign in December 2020. It also illustrates the detection principle of the camera system. The camera records stars as a point source and LEO objects as a streak due to their larger angular velocity. The streak recorded in the picture is the International Space Station (ISS) and the bright object in the center is the full moon.

## Performance estimation

A passive optical sensor benefits from the fact that the sun illuminates the RSO and the reflected light from its surface can be detected via a camera. To detect this signal, the background illumination at the ground-based sensor needs to be low. A clear sky at night is required for successful operation. Furthermore, observations are not possible if the RSO is in the Earth shadow. Which is why under certain observation conditions RSO are not detectable around midnight. This, for example, is the case for zenith line-of-sight (LOS) in winter on the norther hemisphere.

To model the system performance, a spherical RSO is assumed which is illuminated by the Sun. Using the modeled signal-to-noise ratio (SNR) allows to calculate the RSO diameter for a set of system parameters. This gives the minimum detectable RSO diameter d_RSO_ and is calculated as follows [9],

1 $${d_{{{\text{RSO}}}} = \sqrt{\frac{{4\omega _{{\text{R}}} {f^{\prime}}R_{{{\text{RSO}}}}^{{\text{2}}} {\text{SNR}} \sqrt {\frac{{D_{{{\text{opt}}}}^{{\text{2}}} \tau _{{{\text{optic}}}} y_{{{\text{px}}}}^{{\text{2}}} t_{{{\text{exp}}}} \pi L_{{\text{b}}} {\text{QE}} }}{{D_{{{\text{opt}}}}^{{\text{2}}} {\text{ + 4}}{f^{\prime}}^{2} }}{\text{ + }}e_{{{\text{read}}}}^{{\text{2}}} } }}{{{\text{2}}.{\text{755}} \cdot {\text{10}}^{{{\text{21}}}} P\left( {\rho ,\psi } \right)\tau _{{{\text{atm}}}} D_{{{\text{opt}}}}^{{\text{2}}} \pi \tau _{{{\text{opt}}}} y_{{{\text{px}}}} {\text{QE}} }} }.}$$

where *y*_px_ is the pixel size, *f*’ the focal length, *D*_*o*pt_ the diameter of the aperture, and *τ*_opt_ the transmission of the optical system. Furthermore, *τ*_atm_ is the transmission of the atmosphere, QE the quantum efficiency of the detector, SNR the algorithm required signal-to-noise ratio, *e*_read_ the read noise per pixel, *L*_b_ the background illumination, t_exp_ the exposure time, *ω*_R_ the angular velocity of the RSO, R_RSO_ the slant range to the RSO and *P*(*ρ*, *ψ*) the phase function of the RSO. Please see reference [9] for more details.

A more detailed analysis of theoretical system performance parameters of small telescopes for detection and tracking are described in reference [10]. These include the positional accuracy of object tracking, the limiting magnitude, and the information rate of the system. Design parameters are defined which describe optimal system performance. The defined metric for system FOV is the instantaneous field of regard, IFOR, and is defined as,

2 $${\text{IFOR}} = \left( {\frac{{y_{{{\text{px}}}} }}{{f^{\prime}}}} \right)^{2} .$$

where a larger value follows a larger FOV and therefore covers a larger orbital volume. The limiting magnitude m_v_ of such a system is defined as,

3 $$m_{v} = - 2.5{\text{ log}}_{{10}} \left( {\frac{{{\text{SNR }}\sqrt{\sqrt {m_{{{\text{RSO}}}} } {\text{ }}\omega _{{\text{R}}} {\text{ }}f^{\prime}{\text{ }}\left( {e_{{\text{b}}} {\text{+}}e_{{{\text{read}}}} } \right)}}}{{\Phi _{{\text{0}}} \tau _{{{\text{atm}}}} \tau _{{{\text{opt}}}} \frac{{\pi {\text{D}}^{{\text{2}}} }}{{\text{4}}}{\text{QE}}\sqrt {y_{{{\text{px}}}} } }}} \right).$$

where *Φ*_0_ is the Irradiance of magnitude zero object, *m*_RSO_ the number of pixels occupied by the RSO, and *e*_b_ the electrons by the background sky irradiance per pixel.

The information rate shows that the detection performance does not only depend on the sensitivity but also on the FOV, pixel scale and number of crossing RSO. This metric will not be analyzed in more detail but shows the dependencies of the system performance in an analytical way. The metric for information rate is depending on the density of RSO per deg^2^ n_RSO_, the FOV, the information objective J_I_, and the probability of successful RSO detection *P*(*Γ*_RSO+*n*_ > SNR *σ*_*n*_):

4 $$J_{{\text{F}}} = \frac{{n_{{{\text{RSO}}}} {\text{ FOV }}J_{{\text{I}}} {\text{ }}P\left( {\Gamma _{{{\text{RSO}} + n}} {\text{ > SNR }}\sigma _{n} } \right)}}{{t_{{{\text{exp}}}} }},$$

where the information objective *J*_I_ is defined as,

5 $$J_{{\text{I}}} = {\text{log}}_{{10}} \left( {8\left( {\frac{{f^{\prime}}}{{y_{{{\text{px}}}} }}} \right)^{2} } \right).$$

The metric *J*_I_ gives the relative amount of information in a single observation and is inversely proportional to the uncertainties.

Further details about theoretical system performance metrics can be found in ref. [10]. For the system under consideration, Table 1 lists the simulation parameters and results. Due to a small focal length compared to tracking system [10], the information metric is small; therefore, the IFOR is considerably larger and allows observing a larger orbital volume. The minimum detectable RSO diameter is rather small compared to the limiting magnitude of this system.

**Table 1:** Simulation parameters and results of the APPARILLO staring system

Properties	Symbol	Value
Pixel size	*y*_px_	9 µm
Focal length	*f*’	0.2 m
Diameter of the aperture	*D*_opt_	0.1 m
Transmission of the optical system	*τ*_opt_	0.75
Transmission of the atmosphere	*τ*_atm_	0.7
Quantum efficiency	QE	0.55
Signal-to-noise ratio	SNR	4
Read noise per pixel	*e*_read_	15e^−^
Background illumination	*L*_b_	2.38∙10^14^ ph/s/m^2^/sr (17.5 mag/arcsec^2^)
Background electrons	*e*_b_	15000e^−^
Exposure time	*t*_exp_	1 s
Angular velocity of the RSO	*ω*_R_	0.5°/s
Slant range to the RSO	*R*_RSO_	1000 km
Phase function of the RSO	*P*(*ρ*, *ψ*)	0.08
Irradiance of magnitude 0 source	*Φ*_0_	5.6∙10^10^ph/s/m^2^
Number of pixels occupied by the RSO	*m*_RSO_	2px
Results		
Minimum detectable RSO diameter	*d*_RSO_	0.24 m
Limiting magnitude of the optical system	*m*_v_	3.5 mag
Instantaneous field of regard	IFOR	86 arcsec^2^
Information metrics	*J*_I_	9.6

## System details

**Fig. 2:**
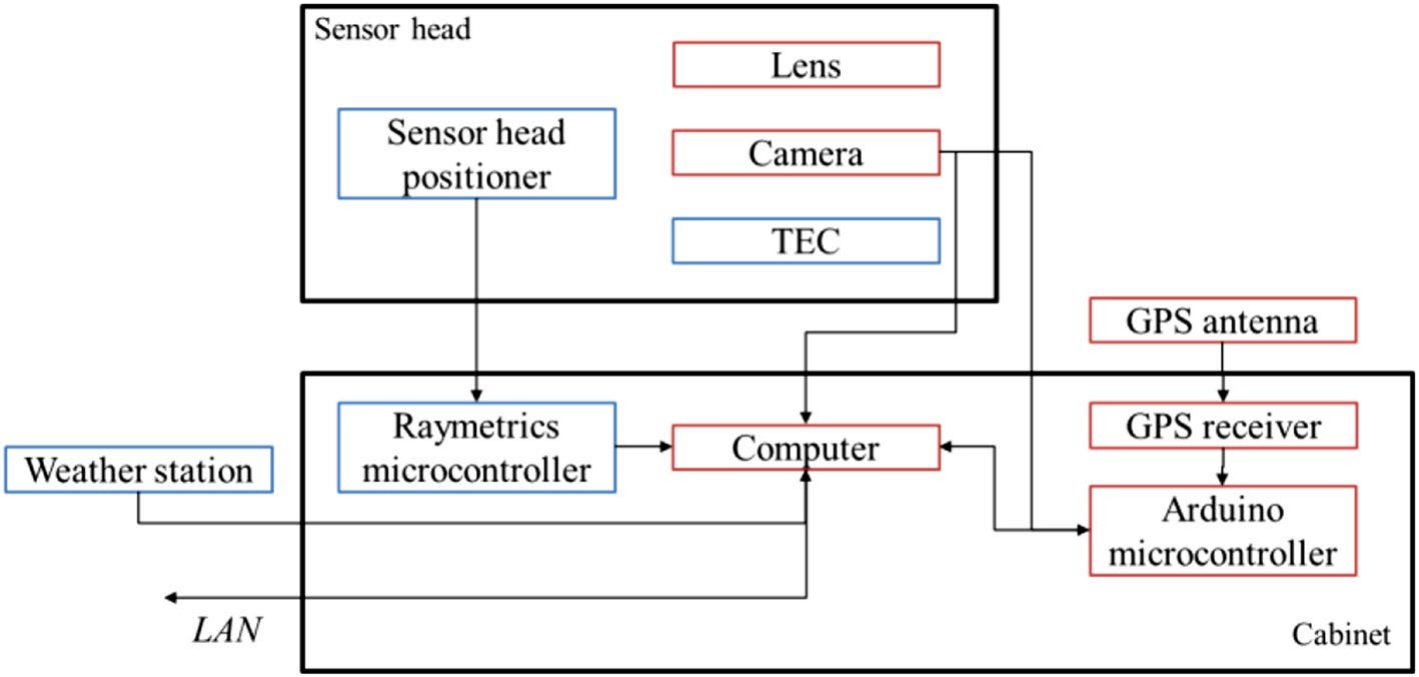
Schematic view of APPARILLO system components and how they are connected. Camera and lens are located in the sensor head and the microcontrollers and PC are located in the cabinet. The core components are marked in red and secondary hardware in blue

The system consists of an imaging camera with a lens, a GPS receiver for time synchronization, a computer for image recording and processing, a weather station, and a weather-proof housing. Latest observations were performed on top of the roof of our office building in Stuttgart Vaihingen. The core system is already operational with a camera, lens and a notebook, which makes it easy to set up and operate almost everywhere. To avoid any maintenance and manual operation, the system was extended with a camera and lens mounting for reliable camera pointing, and a housing for environmental protection. Environmental data measured by the weather station are used to toggle camera acquisition. Figure 2 below shows the location and connection between the components.

### Camera

The system is based on passive optical measurements and the basic components are a camera and a lens to perform angular measurements. As camera we are using a large area CCD (charge coupled device) imaging sensors, namely the FLI PL09000, which has the On Smi KAF-09000 CCD sensor. The large pixel size results in a very good dynamic range of 110,000 e^−^, whereas the readout and dark noise is very low with a total of 15e^−^ per frame. There is also no pattern noise visible, which is typical for CMOS (Complementary metal–oxide–semiconductor) sensors. The image sensor diagonal is 51.7 mm, and the resulting FOV is listed in the next Sect. 3.2. The following Table 2 lists more camera specifications with the settings used for observations.

**Table 2:** Camera specifications and exposure settings used by the APPARILLO system [11, 12]

Properties	FLI PL09000
Sensor	On Semi KAF-09000
Sensor size: width x height, diagonal	36.8 mm × 36.8 mm, 51.7 mm
Pixels	3056 × 3056
Pixel count	9.3 Mpx
Pixel size	12 µm
Full well capacity	110 000e^−^
Typical system noise (@8 MHz read out speed)	15e^−^
Dark noise (@− 35 °C)	0.02e^−^
Shutter	Mechanical blade shutter (Uniblitz CS-65)
Common exposure speed	1 s
Common binning setting	2
Read out rate	8 MHz
Transfer interface	USB 2.0

The downside of these cameras is their relatively slow shutter with a total opening time of 54 ms and closing time of 52 ms [12]. Another downside is the slow image readout and transfer speed, which results from the high resolution in combination with the CCD read out architecture and the USB 2.0 interface. This limits their use to long exposures only and the maximum image recording frequency with our soft- and hardware was 0.2 Hz using binning of 2 (2px × 2px) and 0.1 Hz using full resolution.

### Lens

The tradeoff between different lens choices is between a large aperture and short focal length. A short focal length results in a larger FOV which covers a larger orbital volume. A larger aperture diameter results in a higher sensitivity of the system and therefore better detectability of smaller (fainter) objects.

To keep system size and cost small we decided to use a common single-lens-reflex (SLR) medium telephoto lens. These are commercially available, have a very good image quality across a large image circle (even beyond their designed 43 mm) and are affordable (compared to their alternatives). Table 3 below lists the specifications of lens used during latest observations, including the resulting angular properties with the camera given in previous Sect. 3.1. (see Table 2).

**Table 3:** Lens properties of the lens used during observation campaigns [13]

Properties	Canon EF 200 mm f/2 L IS USM
Bayonet	Canon EF
Focal length	200 mm
f#	2
Aperture diameter	100 mm
FOV: width x height/diagonal (@FLI)	10.4° × 10.4°/14.5°
Pixel scale (@PL09000)	12.4arcsec/px (60µrad/px)

**Fig. 3:**
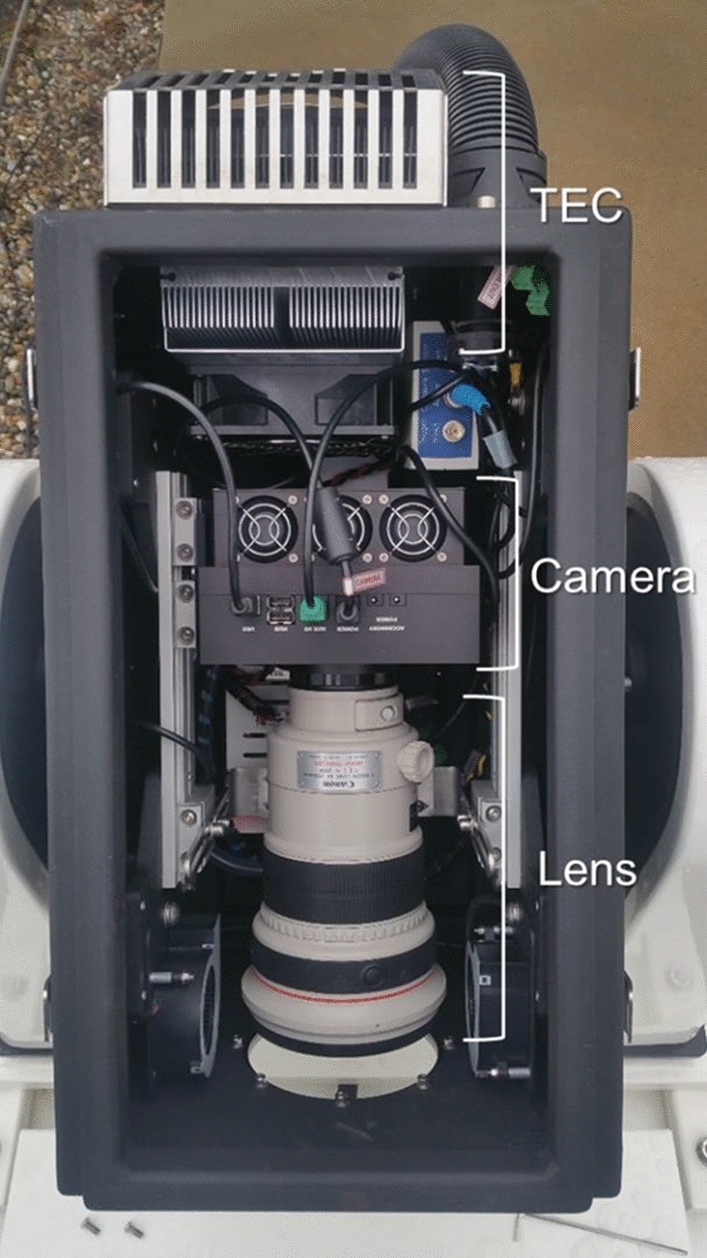
The FLI PL09000 Camera and Canon EF 200 mm f/2 lens are mounted inside the sensor head. A rail on both sides allows mounting the lens and camera. In the back of the head there is the TEC (top of the image) and blowers in front of the window (bottom of the image) prevent it from condensation

A picture of the camera and lens mounted inside the weather-sealed housing can be seen in following Fig. 3 (more details follow in Sect. 3.5.).

**Fig. 4:**
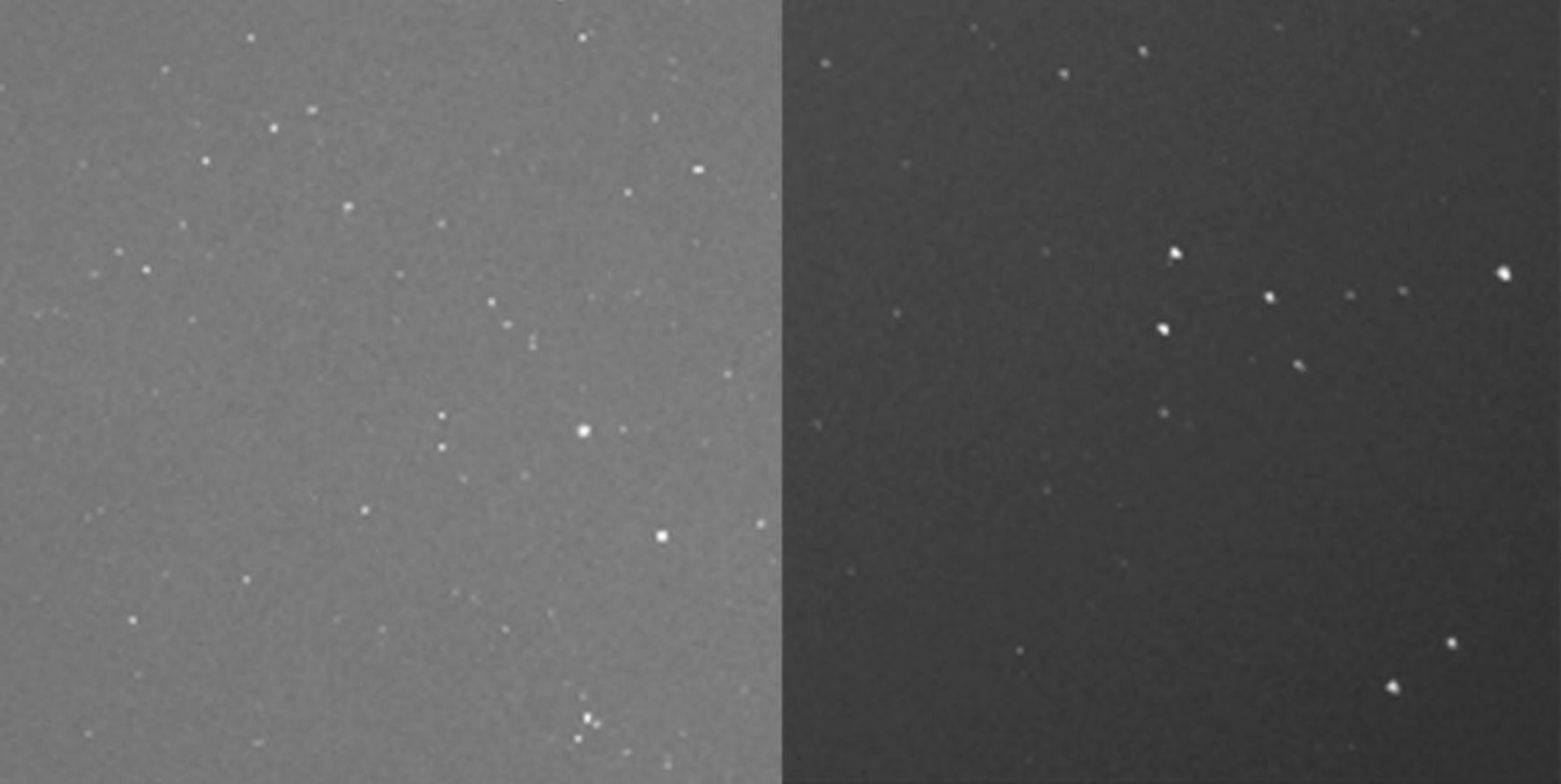
Cropped image of stars recorded by the camera from the center (left) and bottom right corner of the image (right)

For this lens, the image quality is very good across the entire image frame resulting in a point spread function of a star covering only a few pixels, see Fig. 4. The lens show an illumination fall off to the image corners, sample images are shown below (e.g. Figure 6). The vignetting due to the small FOV results mainly from the optical construction where the entrance pupil is obscured by lens element borders, which is typical for lenses with a small focal ratio (f#) [14]. But even without this, vignetting will be present due to the cosine fourth power law. A degradation of the image quality by the window could not be measured/observed, Fig. 4 contains two cropped images, one from the image center and the other from the outer corner of the image. It can be seen that the stars are recorded as symmetric points across the entire image and measure about 1.5px in FWHM.

### GPS synchronization

For GPS synchronization, an Arduino-based GPS timer was developed, it consists of an Adafruit Ultimate GPS receiver and an Arduino Uno microcontroller [15]. It can be used to record the UTC timestamp of an incoming TTL pulse from the camera. The microcontroller compares the incoming TTL pulse from the camera with the PPS signal provided by the GPS receiver. This way, it measures the time of a TTL signal with 100 µs of precision [16].

### Weather station

As weather station the Diffraction Limited, Boltwood Cloudsensor II was used. The weather station records the temperature difference between sky (using an infrared sensor) and ambient temperature, the light level, and if it is raining. This information is used to verify clear sky, darkness, and absence of precipitation, respectively. Following conditions are calculated from the weather data:

- Clear sky: is True, if difference between sky and ambient temperature is smaller than < − 28 K.
- Darkness: is True, if light level is smaller than 10 a.u.
- Dry: is True, if rain equals 0.

### Weather-proofed housing

The weather-proofed housing was developed by Raymetrics^1^ and is an adaption of a wind LIDAR system. Its IP68 rating protects the equipment from the environment. The head is weather-sealed and has a viewing window. Blowers in front of the window as well as a thermoelectric cooler (TEC) prevent condensation on the window and controls the temperature and humidity, this ensures that each component runs within their specified operating conditions. The sensor head contains the camera (shown in Sect. 3.1) and lens (shown in Sect. 3.2) for recording the images, see Fig. 3. The head is movable between 90° and − 90° in respect to the horizon. It points upwards to zenith (or any other suitable elevation angle) during observation and moves the head pointing downwards when the image recording is stopped. This protects the viewing window from any precipitation and the sun accidently being focused on the image sensor or shutter blades of the camera.

**Fig. 5:**
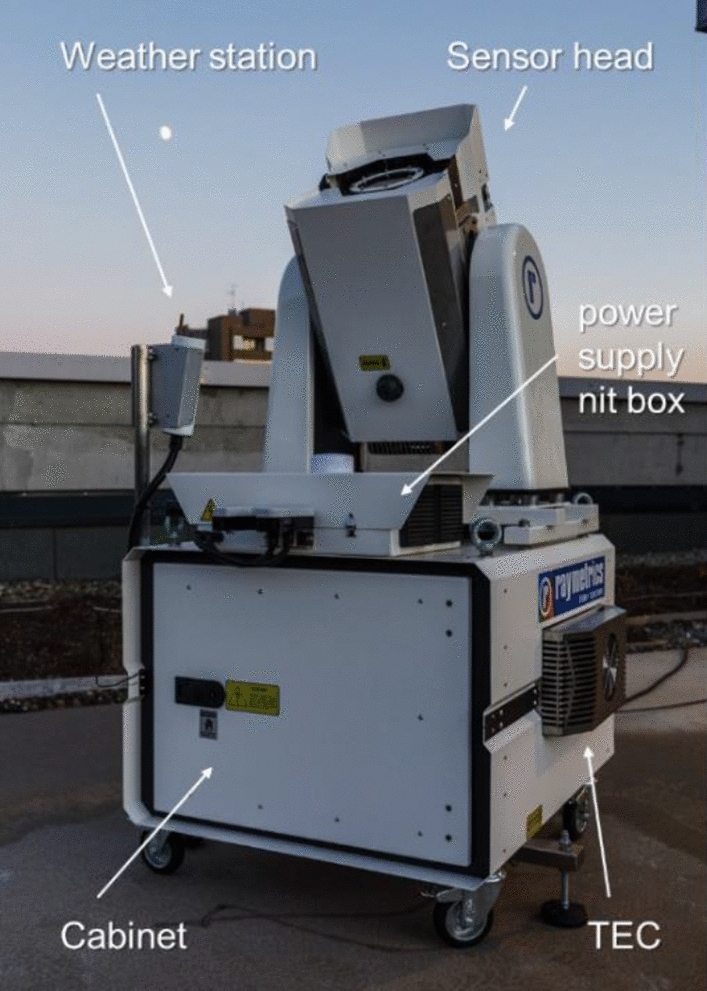
Image of the Raymetrics enclosure during observation just after sunset

The main compartment or cabinet is the control unit, which contains the main controller of the enclosure (SMU200), a 1U UPS, and a power distribution panel. These are mounted on a sliding 19″ rack with a 4U free space to accommodate our workstation computer. On top of the enclosure are mounted the Boltwood weather station, GPS antenna, and power supply nit box (which provides DC current to the electronics). Similarly, the enclosure on the head is environmentally sealed and temperature-controlled. Figure 5 shows the housing and its components with the sensor head pointing upwards.

### Image recording

Image recording is performed with OOOS (Orbital Objects Observation Software), it has been developed by our department for satellite laser ranging (SLR) activities and is highly modular [17]. The software records the images taken by the camera, includes information about observation location, observation line-of-sight (LOS), UTC timestamp from our GPS image timer and meta-data of the camera settings and optics used.

**Fig. 6:**
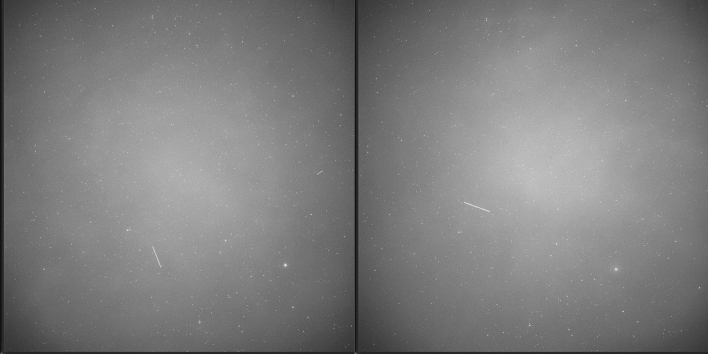
Two-sample images of the APPARILLO staring sensor taken during observations on December 11th at 4:50 am and 5 am

A module named staring daemon handles the automatic image acquisition depending on data provided by the weather station and current time. The setup without any optical filters in front of the lens allows observations with a sun elevation of about -6° and lower to the horizon. This time is automatically calculated and image acquisition is started or stopped depending on the time only if no weather data are available. The sensor head is moved upwards pointing towards the sky and image acquisition is started, if all of the weather conditions (shown in Sect. 3.4) and time constrains are satisfied. If one of the conditions is not satisfied, the image acquisition is stopped and the head moves downwards. If the weather station data are not available, all conditions are considered as satisfied and image recoding is started and stopped based on the computers system time only. It requires the image processing to handle bad images. More on image processing, astrometric calibration and TDM export of tracklets will be described in following chapter 3. When image recording is started by the sensor, astrometric calibration is performed regularly to determine the exact pointing direction of camera system. Typical sample images are shown in Fig. 6.

## Software structure

The image recoding is managed by the so-called Staring Daemon, which is a Python 3 program based on the OOOS software package [17]. The Staring Daemon handles the connected hardware:

- Weather station,
- GPS Timer (Arduino microcontroller),
- Camera,
- Enclosure (Raymetrics),

starts the image acquisition and handles the data export.

### Staring Daemon

**Fig. 7:**
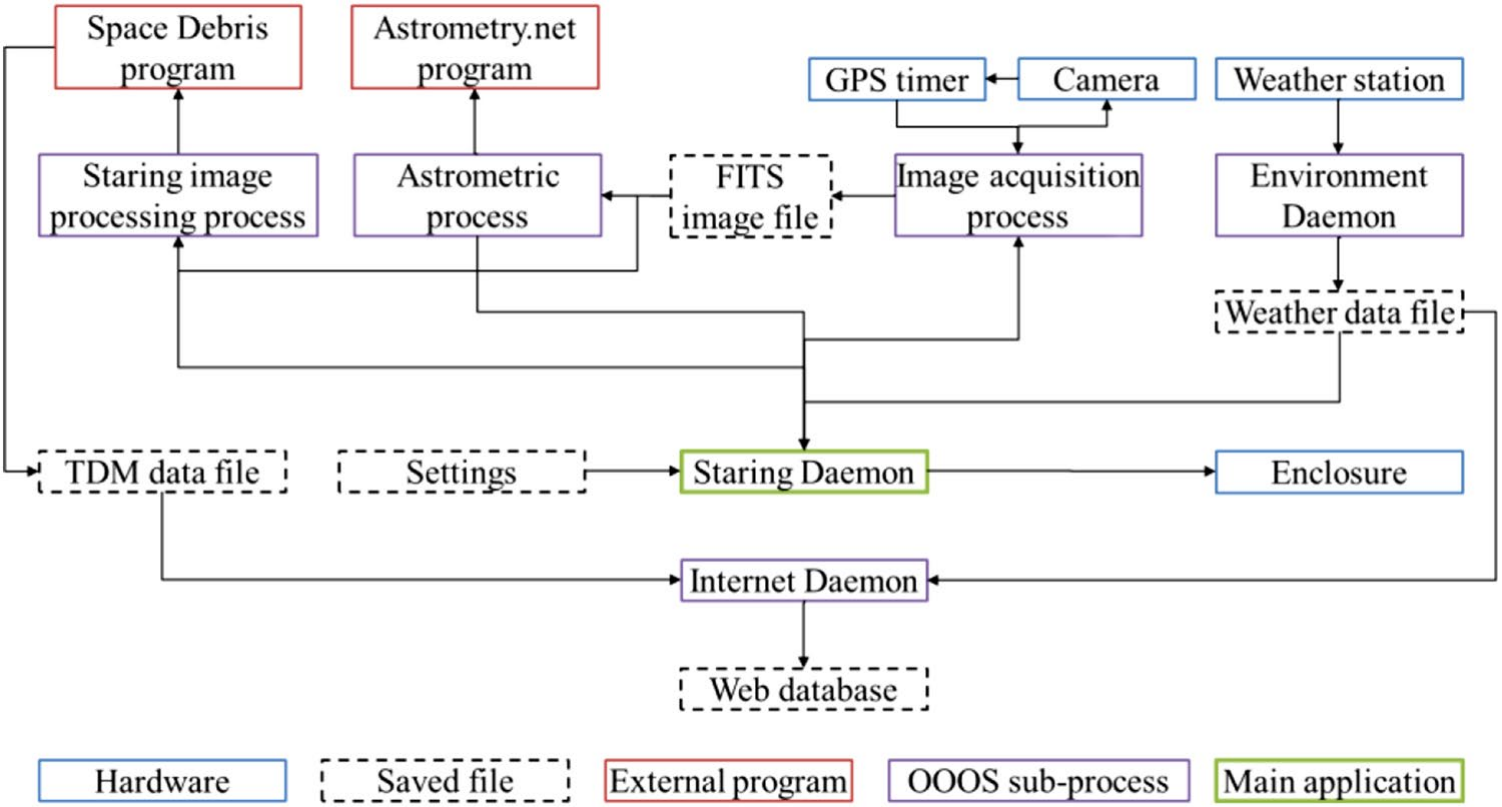
A schematic illustration how the different sub-processes (purple) and hardware interfaces (blue) are connected to the main program “Staring Daemon” (green). Data in- or output is shown in dashed lines and external software programs are marked in (red)

All parts of the software are separated, especially the hardware interfaces are worth mentioning as this follows that the software is not hardware-bound, allowing the user or system designer to select hardware independently. The weather station is controlled by the Environment Daemon and records the weather information. The camera is controlled by the Acquisition Process, which is also connected to the GPS timer. The Staring Daemon itself is connected to those processes and daemons using high-level commands. It reads the recorded weather data and toggles the image recording and sensor head position depending on the weather conditions and time. The Staring Daemon also handles the communication to the image processing program and hands over information and measurements to be uploaded to our website by the Internet Daemon. Following Fig. 7 gives an overview of the external programs (Space Debris [18], Astrometry.net [20]), OOOS Daemons, OOOS processes and data transfer between those.

### Image processing

**Fig. 8:**
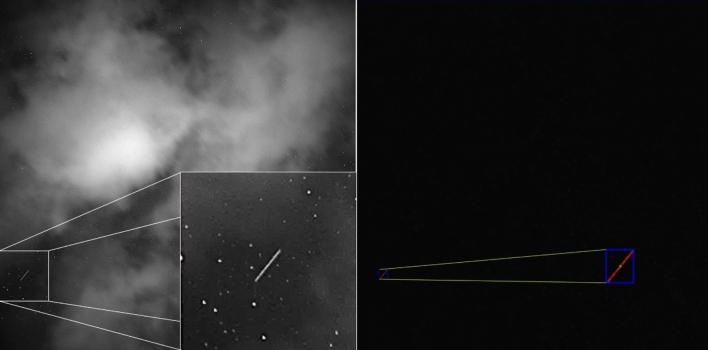
Sample image of the staring sensor of partly cloudy sky (left). The software is designed to handle clouds and still detect orbital objects recorded as streaks (right). As long as there is a dozen of stars detectable to perform astrometric calibration, the measured streak position can be converted to equatorial coordinates

Image processing is performed by a separate program written in C++. The software was developed in cooperation with Kormoran Technologie GmbH^2^ and is simply called “Space Debris” [18]. It reads in the recorded FITS image files and uses OpenCV [19] algorithms to process the images. A major challenge for the processing is the presence of clouds in the images, see Fig. 8 below. Combined with stray light from artificial light sources, this caused too many false-positive detections previously [3]. For autonomous operation, the new software was included and false-positive detection could be rejected completely during our campaigns in December 2020.

To detect objects in the images, the background intensity profile is determined by filtering high frequencies from the image first. The result is subtracted from the original image to remove the background. This process is performed iteratively which removes clouds or other intensity variations. The image can be binarized to separate the objects (e.g. stars or streaks) from the background. Stars and streaks are distinct by their size and shape. Pixel coordinates of the stars and streaks are measured subsequently. The star positions are used to perform astrometric calibration, which allows converting the streak coordinates into equatorial coordinates. As astrometric software the engine of the Astrometry.net project is used [20], installed as a separate program. Up to this stage, all calculations are done separately for each image.

### Data export

In the next step, the equatorial coordinates of the streaks observed in several different images are combined into traces by the angular velocity and direction in the sky. Finally, a straight line is fitted with constant velocity into the observed data. This way we obtain a circular fit of the measured coordinates to remove any outliers. The results are written into a “Tracking Data Message” (TDM) file [6] which allows data sharing with other stations or databases. These unidentified TDM files are uploaded directly to our web database using the Internet Daemon, which delivers the connection for interchanging the data to a separate subsystem. Chapter 4 shows the resulting data of the first unsupervised campaign of the fully autonomous staring system.

## Results

Compared to manual operation [3], the system now can use every minute to observe when conditions turn good. We operated the system constantly between November 20th and December 23rd. The weather conditions covered classical German winter weather, including storm, rain, fog, frost and snow. The conditions were far from optimal, making it a worst-case scenario for the system. During the campaign, APPARILLO was placed on top of the roof of our office building as shown in Fig. 1. The observation direction was fixed in the horizontal reference frame over the entire campaign. Zenith was chosen as LOS because it should give the best performance [9]. The system settings, properties and geodetic coordinates are listed in Table 4.

**Table 4:** APPARILLO configuration and settings during December 2020 campaign (20.12.2020–23.12.2020). The LOS was determined by astrometric calibration and the geodetic position derived from the GPS timer

Parameter	Value
Camera	FLI PL09000 (see Table 2)
Lens	Canon EF 200 mm f/2 L IS USM (see Table 3)
FOV	10.4° × 10.4°
Diameter of aperture	0.2 m
Exposure time	1 s
Frame rate	0.2 Hz
Binning	2
Pixel scale	0.07°/px (24.8arcsec/px or 120µrad/px)
Line-of-sight (LOS)	
Azimuth	144°
Elevation	88°
Geodetic coordinates	
Latitude	48.74885° ± 5e-05°
Longitude	9.10257° ± 4e-05°
Altitude	486 m ± 6 m

**Fig. 9:**
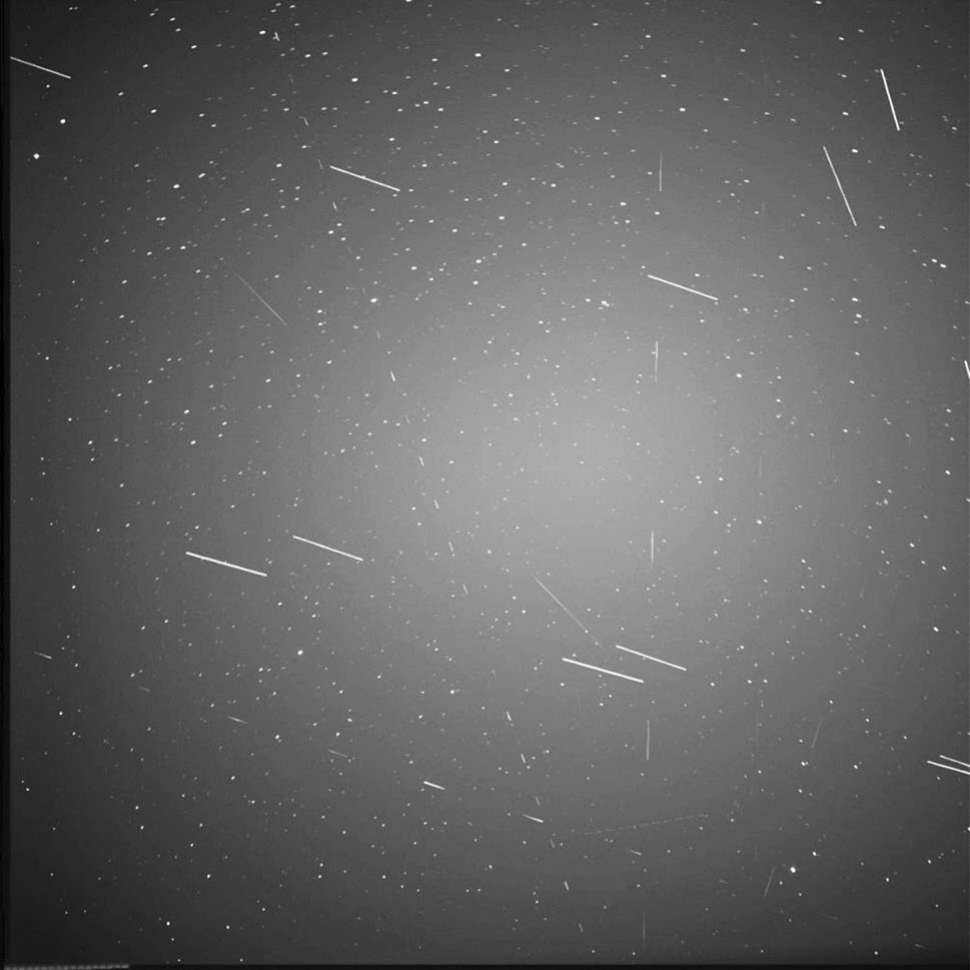
89 images merged to show objects detected by the staring system on November 26th. 10 Objects passed the FOV over a duration of 13.5 min between 04:57:18 and 05:04:44

Figure 9 shows a series of 89 images combined to show how streaks are recorded by the system. It contains low altitude LEO RSO as long streaks, high-altitude LEO RSO as short streaks and rotating RSO with visible intensity variations.

**Fig. 10:**
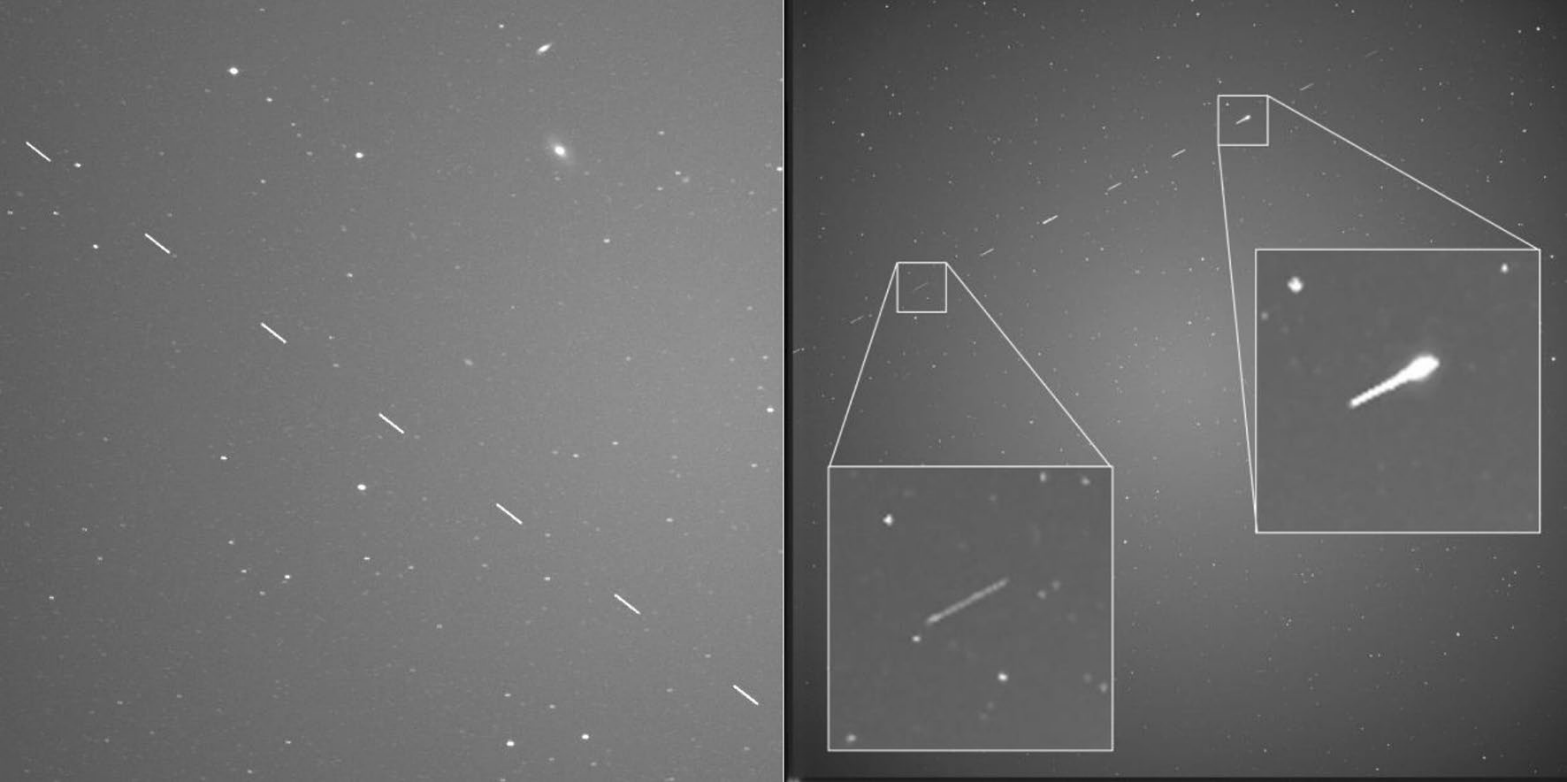
Left: Cropped image showing SL-12 Rocket body (NORAD: 24829) as streaks passing right in front of the galaxies M81 and M82 (upper right), 8 imaged were merged taken on 2020-12-17 between 03:09:00:569 and 03:09:36:601. Right: Intensity variations recorded from GLOBALSTAR M015 (NORAD = 25308), here 12 images were merged recorded on 2020-11-26 between 03:29:21 and 03:30:02

Two more samples of a combined series of images containing a single object in the FOV are shown Fig. 10 below.

**Fig. 11:**
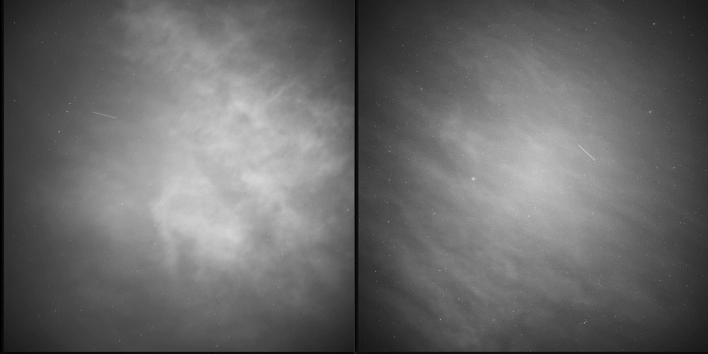
Two images showing a high cloud layer and a LEO RSO in each image. Left: STARLINK-1312, (NORAD = 45398) at December 17, 2020 06:12:06:970. Right: USA 25 (NORAD = 18025) at December 11, 2020 05:52:39:514

The cloud sensor does not prevent the occurrence of clouds in the images. Especially transparent high clouds were often recorded, see Fig. 11.

The image processing provides a lot of parameters to be adjusted which affects sensitivity, false-negative and false-positive detection rates. The settings are chosen to have zero percent false-positive detections. During latest campaign, this requirement was fulfilled, but due to very few air traffic during that time (caused by the COVID-19 pandemic), no aircraft crossed the LOS. This is to current knowledge the only case the system might falsely detect an RSO.

However, false-negative detections were caused from faint objects or rotating RSO with large-intensity variations (like shown in Fig. 10, right). These intensity variations result in higher momentum of the grayscale streak which causes a false-rejection. This kind of exclusion is implemented because of background stars that coincide with the streak induce misplaced detections. Currently, we cannot provide an exact number of the actual false-negative rate as the human observer shows large variations in detecting streaks from a stack of images which range up to 8500 images per night. In a manual review of 15% of the data, a false detection rate of 4% was observed. These were the number of streaks which a human could detect, but not the image processing. It should be noted that the human on the other hand missed about the same number of streaks which the software did detect properly.

For each detected object, the corresponding images were reviewed to validate the measurements. Next, the recorded TDM data are analyzed to review the performance of the system in more detail.

### Detection statistics

During the campaign, the system was running continuously without any interruptions. The following Table 5 lists how many objects (TDM files) were recorded each night. Furthermore, the amount of images recorded, the number of identified objects using the CelesTrak TLE catalog (more details follow in the end of this section) and the resulting unidentified objects are listed. An object was correlated with the available TLE predictions [4] and considered as identified when all measured coordinates divert less than 1° from the prediction. Prediction uncertainties of TLE have been reported being as large as 517 m for in-track and 137 m as average residuals across the catalog for LEO [21]. Considering a 3σ limit, this results in a maximum angular radius of 0.15°.

**Table 5:** Identification statistics of detected objects during observation campaign in December 2020, see Table 4 for system settings

Date	Detected RSO (TDMs)	Identified TLE RSO	Unidentified RSO	Percentage of unidentified RSO (%)	Images
20.11.2020	40	31	9	22.50	3866
21.11.2020	0	0	0	0.00	0
22.11.2020	0	0	0	0.00	0
23.11.2020	17	17	0	0.00	1517
24.11.2020	0	0	0	0.00	189
25.11.2020	64	55	9	14.06	7868
26.11.2020	60	52	8	13.33	6496
27.11.2020	33	22	11	33.33	3396
28.11.2020	52	42	10	19.23	2455
29.11.2020	26	21	5	19.23	5399
30.11.2020	42	31	11	26.19	4755
01.12.2020	0	0	0	0.00	42
02.12.2020	0	0	0	0.00	3
03.12.2020	65	60	5	7.69	2158
04.12.2020	0	0	0	0.00	2548
05.12.2020	0	0	0	0.00	2301
06.12.2020	0	0	0	0.00	4270
07.12.2020	6	5	1	16.67	1218
08.12.2020	3	2	1	33.33	409
09.12.2020	0	0	0	0.00	137
10.12.2020	43	39	4	9.30	1052
11.12.2020	0	0	0	0.00	252
12.12.2020	0	0	0	0.00	5
13.12.2020	69	60	9	13.04	5516
14.12.2020	1	0	1	100.00	1274
15.12.2020	4	2	2	50.00	1628
16.12.2020	133	114	19	14.29	7369
17.12.2020	3	3	0	0.00	3893
18.12.2020	138	117	21	15.22	8546
19.12.2020	7	7	0	0.00	317
20.12.2020	20	20	0	0.00	1906
21.12.2020	0	0	0	0.00	69
22.12.2020	5	4	1	20.00	1892
23.12.2020	0	0	0	0.00	74
Sum	831	704	127	15.28	82,820

**Fig. 12:**
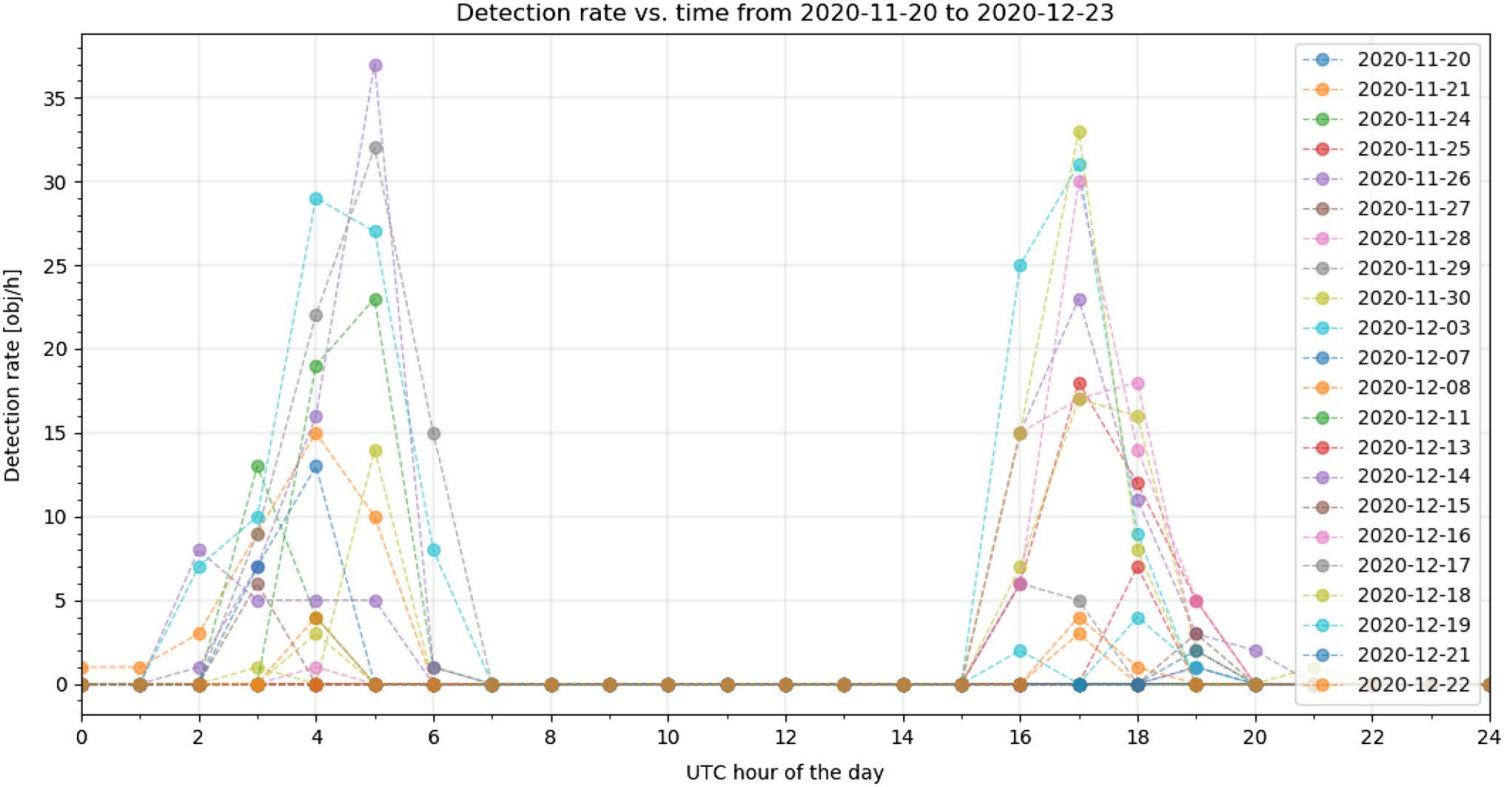
Hourly detection rate of all observations between November 20th and December 23rd 2020, see Table 4 for system settings. Days without any detections are excluded

Compared to former observation campaigns from 2015 [3], the number of unidentified objects has been reduced by about 1/3 (15% vs. 23%). Former observations were performed manually during good conditions only. These conditions were: good weather when the night sky was clear and when LEO RSO are illumined by the sun while the observer is in the earth’s shadow (at night). This is about 1 h after sunset for 4 h and 5 h before sunrise to 1 h before sunrise. Current observations are performed across the entire night depending on the weather conditions, which results in no detection around midnight but lot of detections after sunset and before sunrise. Following Fig. 12. shows the hourly detection rate of all nights between November 20th to December 23rd. Days without any detections are excluded. The detection rate is shown over the time of the day in 1-h steps.

**Fig. 13:**
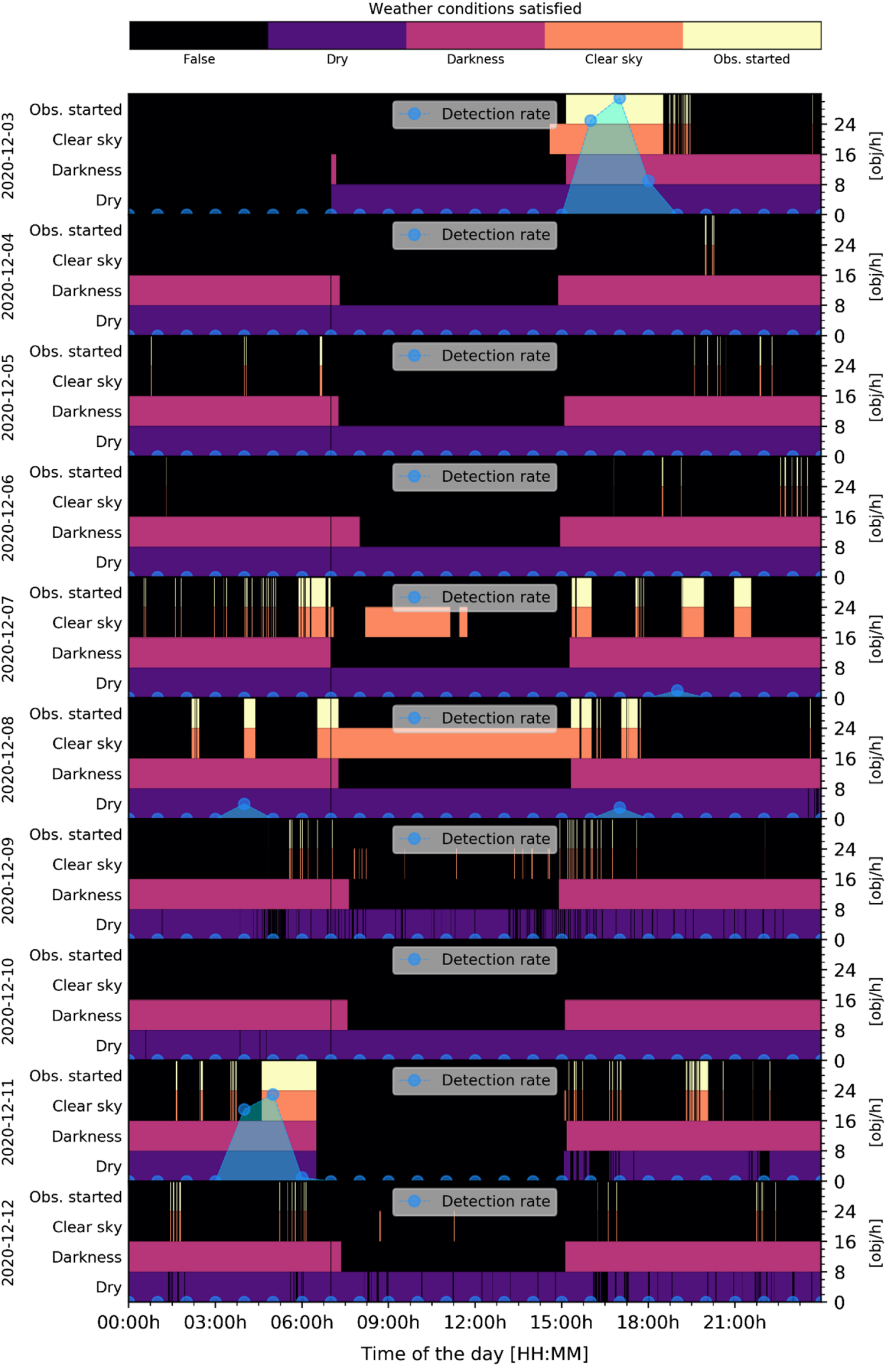
Measured weather conditions and the corresponding detection rate of the APPARILLO system between December 3rd to 12th. Each of the 10 days is shown in a separate row. Within each the conditions: Dry (purple), Darkness (magenta), Clear sky (orange) and Observation started (yellow) are shown in four separate rows over the UTC time of the day. If any condition is not satisfying it is shown in black. On the right axis, the hourly detection rate is shown in blue over the time of the day, see Table 4 for system settings

**Fig. 14:**
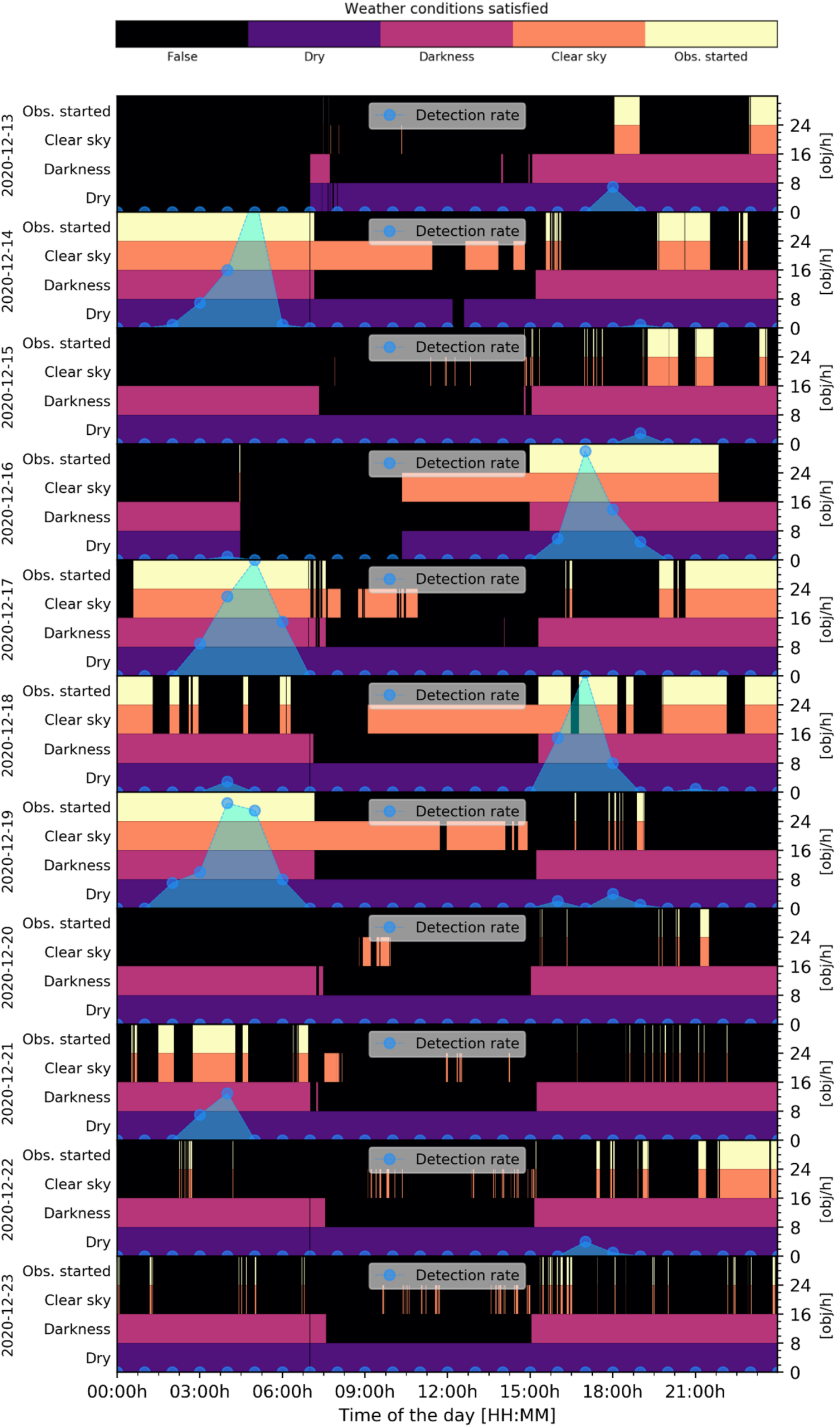
Measured weather conditions and the corresponding detection rate of the APPARILLO system between December 13th and 23rd. Each of the 11 days is shown in a separate row. Within each the conditions, Dry (purple), Darkness (magenta), Clear sky (orange) and Observation started (yellow) are shown in four separate rows over the UTC time of the day. If any condition is not satisfying, it is shown in black. On the right axis, the hourly detection rate is shown in blue over the time of the day, see Table 4 for system settings

During good conditions, the detection rate is about 30 objects per hour which is 20% higher than previously measured (24.2 obj/h) [3]. This is due to the larger population of LEO RSO compared to 2015. The largest measured detection rate was 36 objects per hour. It can be seen that the system can only detect object during the terminator phase (during twilight hours). These are between 2 am UTC to 6 am UTC in the morning and 4 pm UTC to 7 pm UTC, round midnight (11 pm UTC), the system cannot detect any RSO. It should be noted that observation conditions were various during the campaign, but the system was capable of recording objects once there was a gap in the clouds, which was the case on December 16th and 18th in the morning or in the afternoon of December 13th, 19th and 22nd. Following Figs. 13, 14 show more details of the measured weather conditions and the corresponding detection rates for each day in a separate graph. In each graph, there are 4 separate rows showing the weather conditions: dry (violet)/rain (black), darkness (magenta)/light (black), clear sky (orange)/cloudy (black) and if the observation was started (yellow). E.g. it shows on which time the observation was started as a yellow bar and the resulting detection rate is shown on the right y-axis in blue depending on the time of that day. The presented data cover only 20 days in this article due to space constrains.

The system performed very reliable in judging the weather conditions and observations were started automatically. When weather conditions were good during the terminator phase, the detection rate went up to 36 objects per hour for the 14th of December but mostly ranged between 25 and 30 objects per hour. The only unexpected behavior, which we observed, was wrong recording of the delta sky to ground temperature when the weather sensor became wet. This can be seen as fine lines (orange) in the Clear sky condition in Figs. 13, 14, which caused the system to start observation falsely for a few minutes. Remarkably, the image processing handled those situations without a single false-positive detection. To get more details on the system performance, detected objects need to be identified. These results are presented in following Sect. 5.2.

### Size of detected objects

**Fig. 15:**
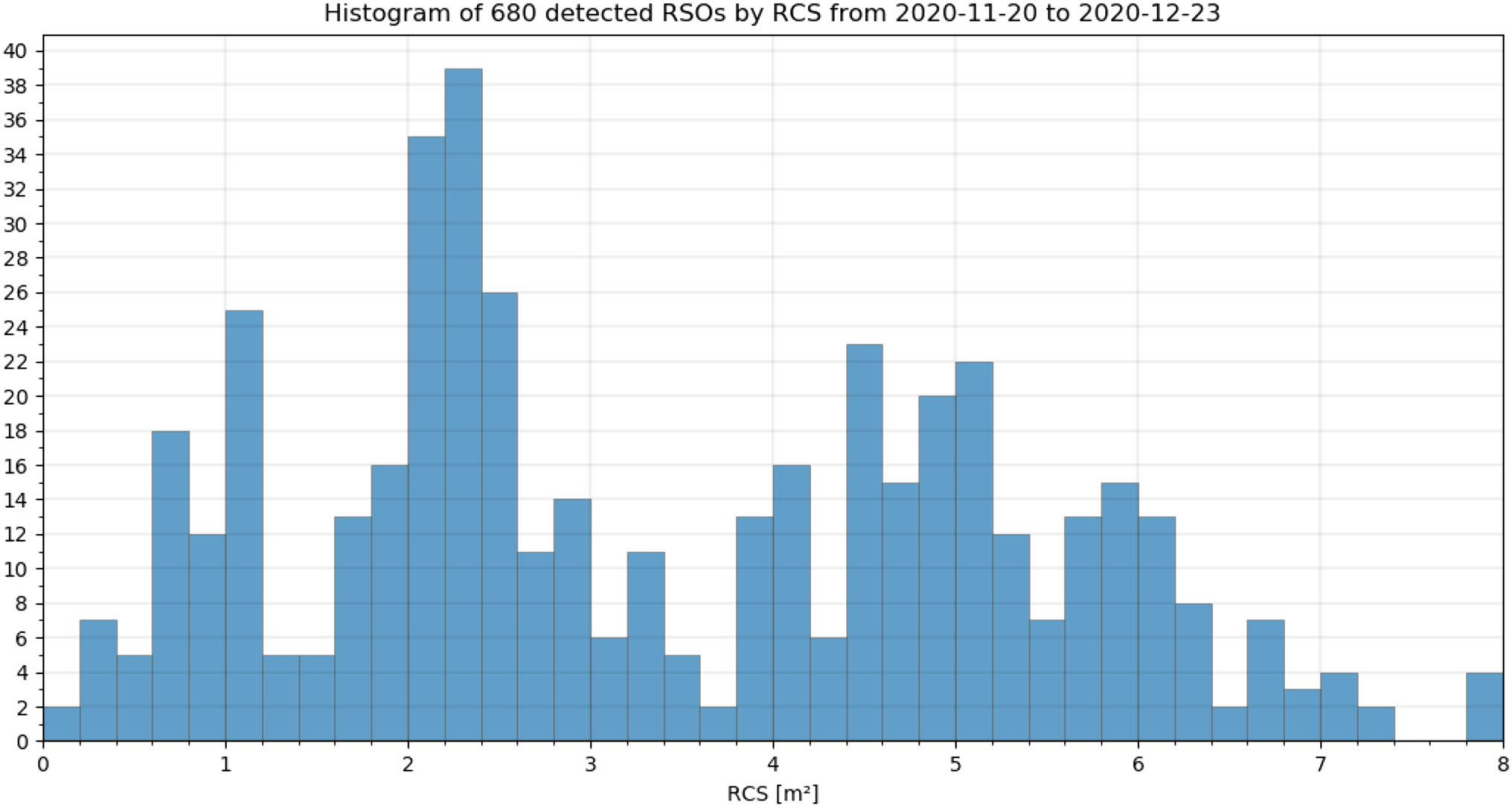
Histogram of detected and identified RSOs grouped by their RCS according to SATCAT [22]. Data taken between November 28th and December 23rd, see Table 4 for system settings

**Fig. 16:**
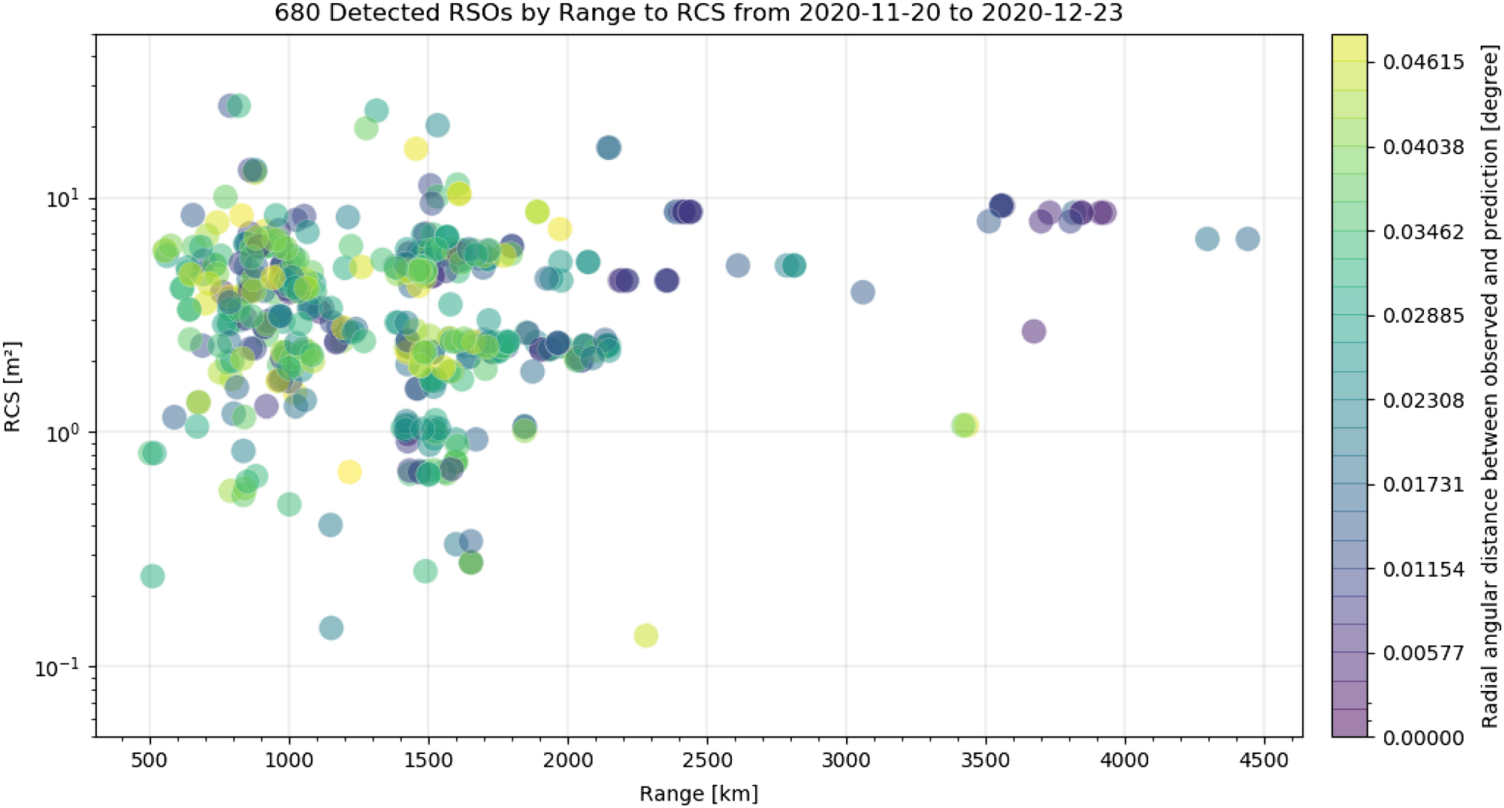
RCS according to SATCAT [22] and range according to TLE predictions [4] of each detected and identified objects as a circle. The color of each circle correlates with the mean radial angular deviation of the angular measurements to the predicted positions (according to TLE prediction) of this detected object. Ranging from zero (purple) to 0.05° (yellow). See Table 4 for system settings

All detected objects were compared with CelesTrak’s TLE [4] and SATCAT (Satellite catalog) [22] catalog. The TLE catalog is operated and maintained by the NORAD (North American Aerospace Defense Command) and contains publicly available predictions of RSOs. The SATCAT catalog contains supplementary information, like RCS (Radar Cross Section) or launch date of those RSO. All 823 detected RSOs are compared with every object in the TLE catalog to identify it and the SATCAT is used to obtain the RCS if available. The SATCAT does not provide information of all 704 detected TLE objects, which reduces the amount of data to be analyzed to 680. The detection statistics grouped by their RCS is shown in following Fig. 15 as a histogram and in relation to the (predicted) objects distance during observation in Fig. 16.

Figure 15 shows that objects with an RCS in the range between 0.1 and 20 m^2^ were detected. And the number of detected objects is peaking around 1 and 2.2 m^2^. As the total number of RSO increases with smaller object size, this indicates that the number of undetected objects rises with objects smaller then RCS 2 m^2^. In Fig. 16, it can be seen that there is no direct correlation between RSOs RCS or range and the radial angular displacement of the measurements to the predicted positions. The residual deviation of the measured angles of a single object also does not correlate with RCS or range (angular velocity). In Sect. 5.3, the angular measurements are analyzed in more detail.

**Fig. 17:**
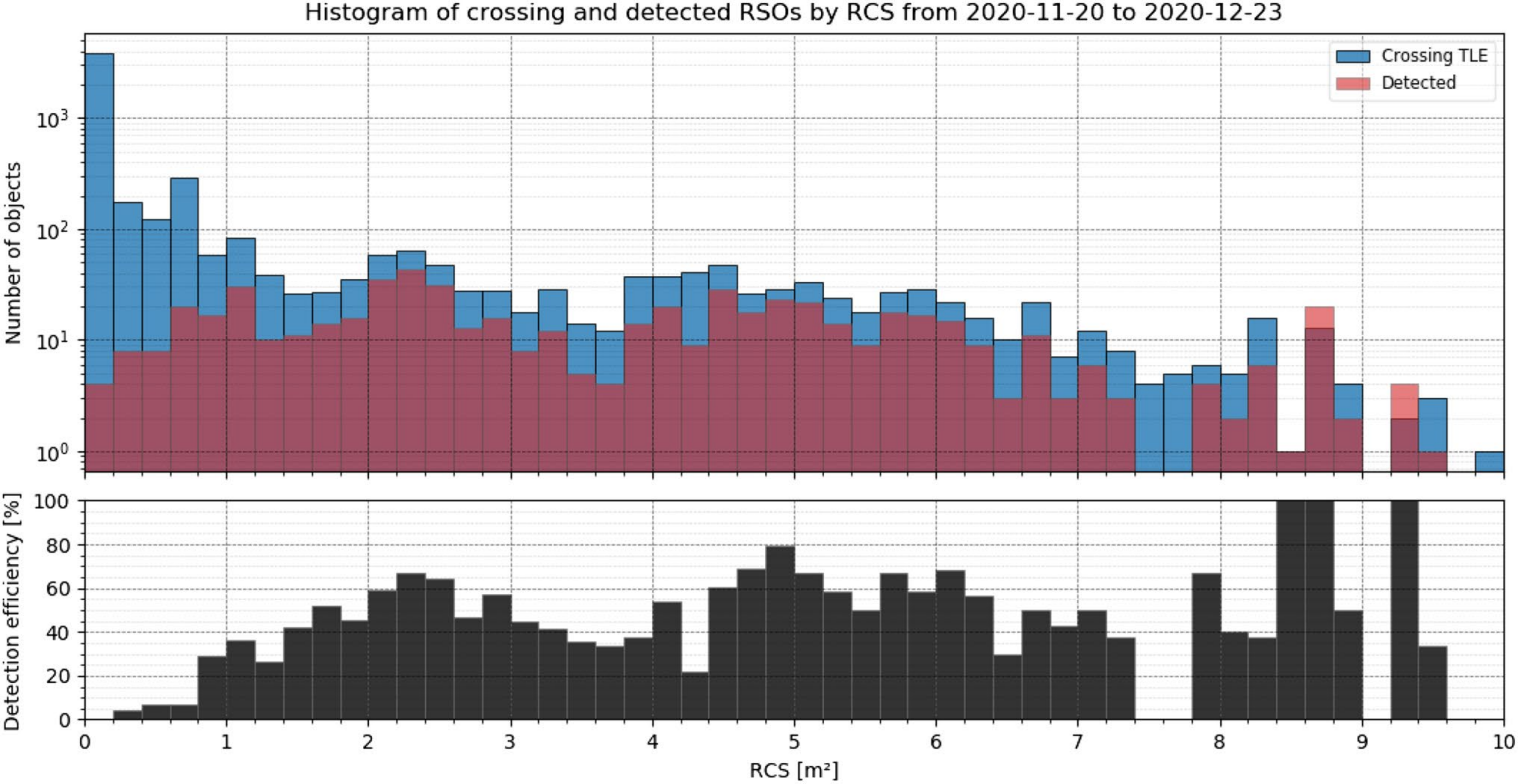
Top: Histogram of detected RSO by APPARILLO (red) and crossing TLE objects (blue) according to CelesTrak NORAD TLE predictions grouped after RCS. The crossing RSOs are calculated when the system performed observations, if no observation was started crossing TLE objects are not considered. Bottom: And the corresponding detection efficiency (black). The objects were recorded between November 20th and December 23rd, see Table 4 for system settings

Furthermore, the detected object can be compared to the cataloged NORAD objects which crossed the FOV during observation. This is also performed using the TLE orbital data and allows to calculate the detection efficiency to all cataloged objects. The detection efficiency gives a figure of merit how good the system can detect certain object size. Following Fig. 17 shows a histogram of detected objects in red and crossed objects in blue according to TLE predictions [4] for different RCS [22]. The bottom half shows the resulting detection efficiency.

Figure 17 shows that the detection efficiency for objects with an RCS larger than 1 m^2^ is about 50%. Even though objects with an RCS as small as 0.1 m^2^ were detected, the detection efficiency is effectively 0% due to the large population of smaller RSO.

**Fig. 18:**
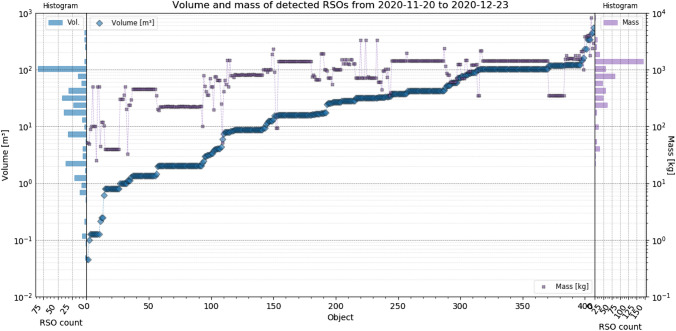
For every object sorted ascending to its volume, the volume (blue) and mass (purple) are shown, according to DISCOS [23]. The corresponding mass distribution is shown on the right *y*-axis and volume distribution on the left *y*-axis. Data are taken between November 20th and December 23rd, see Table 4 for system settings

**Fig. 19:**
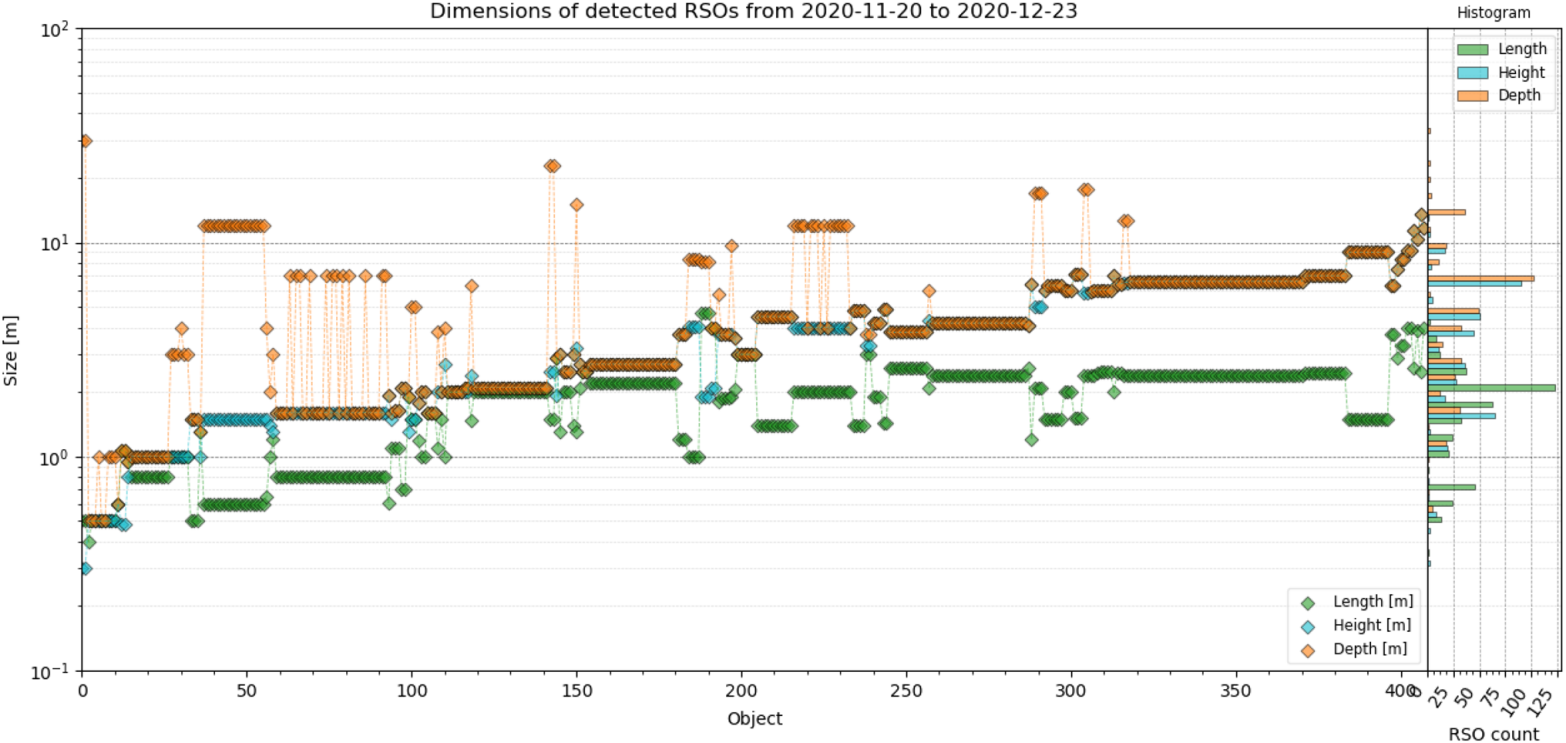
The dimensions of every identified object, sorted ascending to its volume. The RSO length (green), height (cyan) and depth (orange) is shown left and the corresponding distributions of detected objects is shown for each dimension in the same colors on the right *y*-axis. Data are taken between November 20th and December 23rd, see Table 4 for system settings

**Fig. 20:**
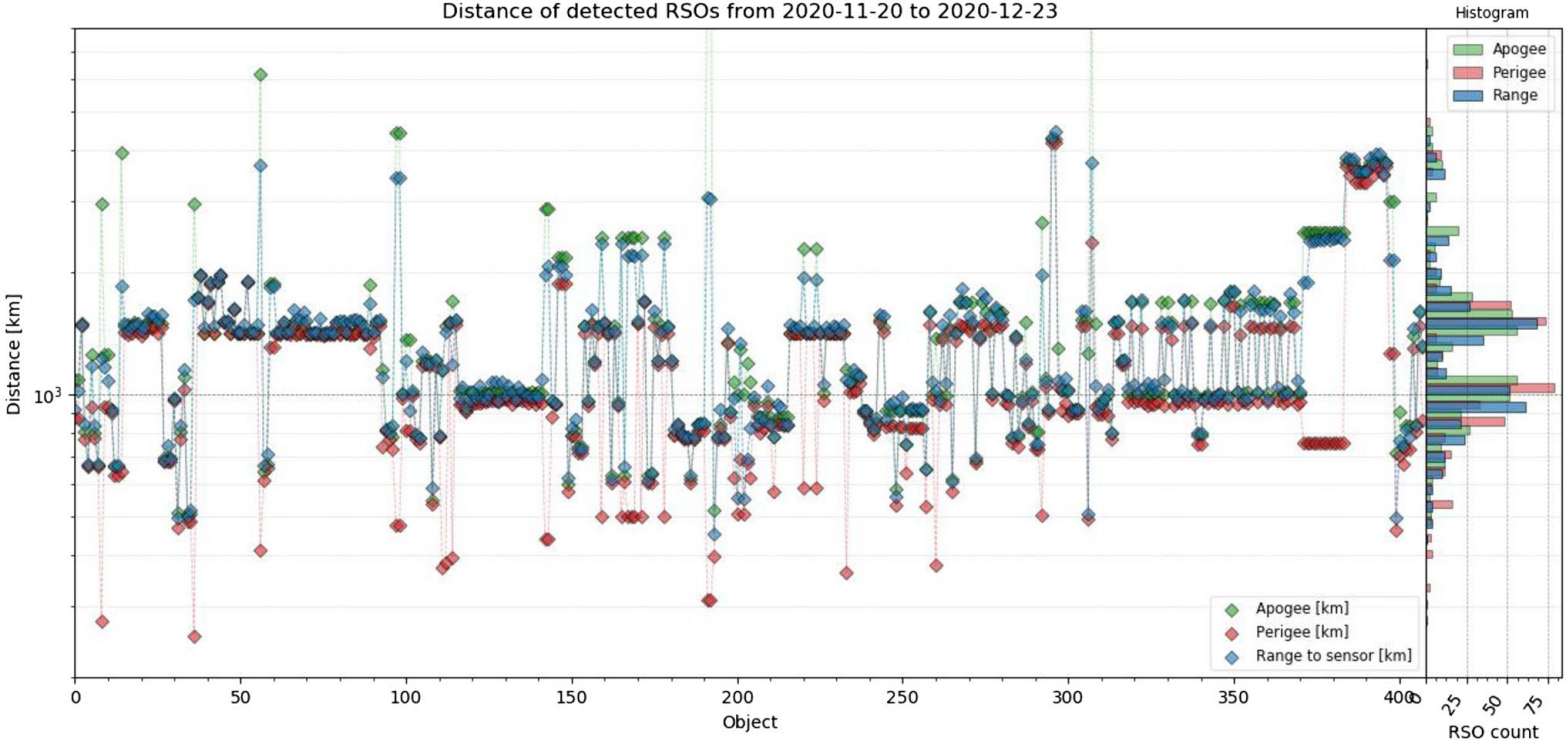
The apogee (green), perigee (red) and range (blue) of very object, sorted ascending to their volume. On the right, the corresponding distribution is shown using the same colors. Data are taken between November 20th and December 23rd, see Table 4 for system settings

**Fig. 21:**
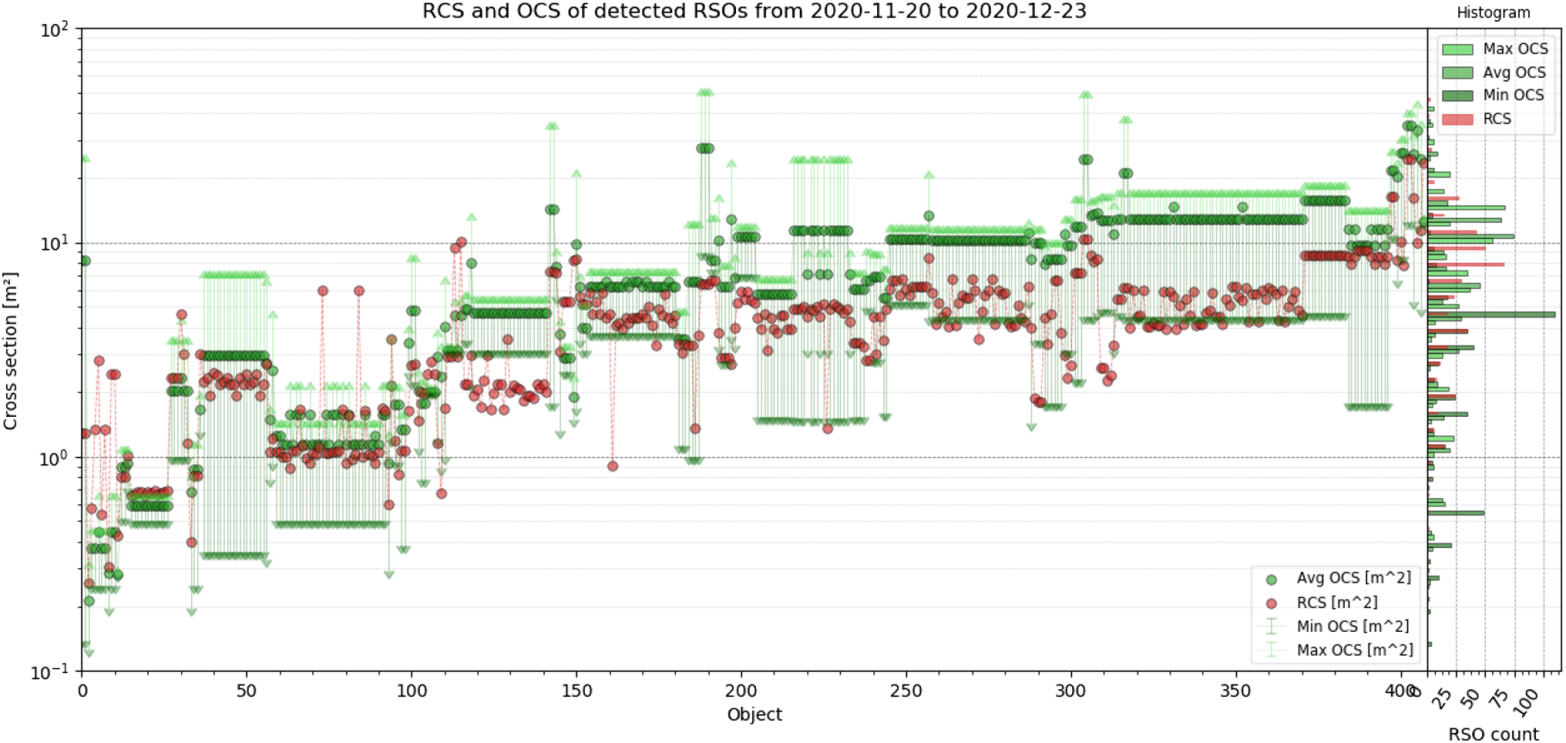
Comparison between RCS (red) obtained from the SATCAT [22] and OCS (green) obtained from DISCOS catalog [23]. The minimum (dark green) and maximum OCS (light green) is shown as an error bar. On the right y-axis, the corresponding distribution of each dataset is shown using the same colors. Data are taken between November 20th and December 23rd, see Table 4 for system settings

ESA’s DISCOS catalog [23] allows to obtain physical properties and optical cross section (OCS) of RSOs. The catalog does not provide data of every identified object, but for 410 RSO, the data were available. This allowed evaluating the system’s detectability to RSO dimensions, OCS and mass. In the following, all objects were sorted ascending to their volume. Figure 18 shows the volume and mass, Fig. 19 the dimensions (length, height, depth), Fig. 20 range, apogee and perigee and Fig. 21 compares the RCS to OCS for each object. Additionally, a histogram shows the distribution of each dataset on the y-axis of the graph.

The detected RSOs range between 40 kg and 4t in mass and between 0.045 and 550 m^3^ in volume. The dimensions of each objects are shown in Fig. 19. Except the smallest objects (Transit 12 (NNS O-8), NORAD 2119, COSPAR: 1966-024A), the dimensions are in the same order of magnitude. As a general rule, the system mostly detects objects with a size of a dish washer and larger.

The majority of detected objects measure a few meters in size. The satellite with the smallest volume measures 30 m in depth. Hence, his OCS is larger than the next larger objects, which measure about 1 m in each dimension.

Following Fig. 20 shows the apogee, perigee and calculated range from the TLE predictions of each identified object, to show if the detectability correlates with the orbit apogee and perigee.

It can be seen that the detected size does not depend on the range to the system. All the objects are detected around 1000 km of range, with a few objects as low as 550 km and up to 4500 km. With a few exceptions the orbits are mostly circular.

The DISCOS catalog also provides a range of OCS values, namely minimum OCS, average OCS and maximum OCS. This represents how the OCS varies as many factors have an impact on the RSO brightness, like phase angle, material, shape, and size. The following Fig. 21 compares the RCS with these three OCS values for each identified object.

The data show that in general, the OCS, RCS, and dimensions are correlated with the OCS being more often larger than the RCS. And the RCS and OCS are similar to the actual dimensions (volume) of each RSO, compare Fig. 21 with Figs. 18, 19. Thus, the RCS statistics shown in Fig. 17 is a good way of illustrating system performance and allows to estimate the dimensions of detected objects.

Next Sect. 5.3 evaluates the angular measurements of the detected and identified objects in more detail.

### Angular measurements

**Fig. 22:**
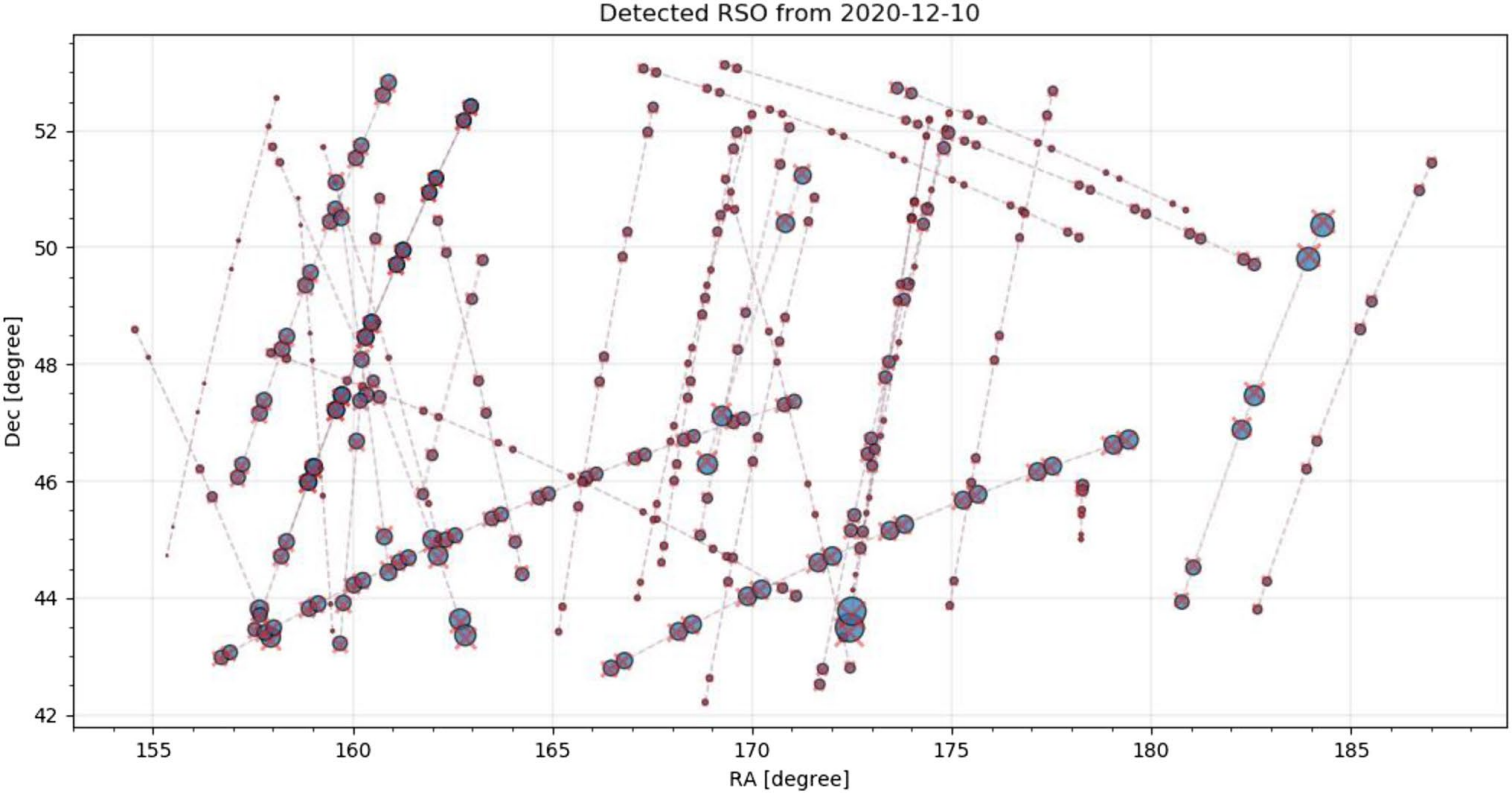
Equatorial positions of detected RSO by the staring system (red cross) and the corresponding TLE object (blue circle). The size of the markers shows the relative angular displacement between measurement and prediction. Data taken on December 10th, see Table 4 for settings

**Fig. 23:**
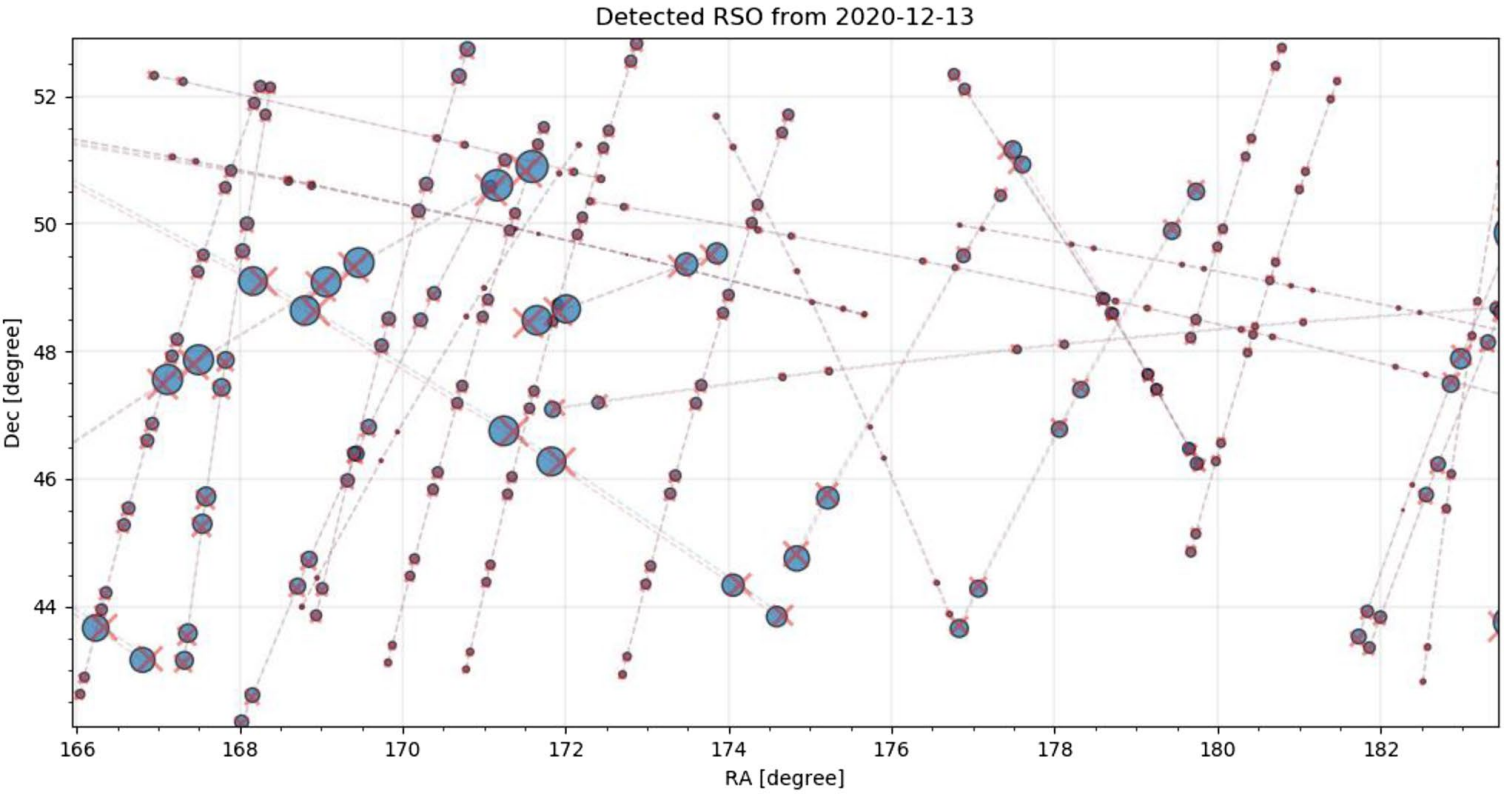
Equatorial positions of detected RSO by the staring system (red cross) and the corresponding TLE object (blue circle). The size of the markers shows the relative angular displacement between measurement and prediction. Data taken on December 13th, see Table 4 for settings

The available TLE prediction data are also used to compare recorded angular position with predictions. Two-sample visualizations from a random subset of detected RSO are shown in Figs. 22, 23. They each show the measured equatorial coordinates of about two dozen RSO and its corresponding predicted position calculated using TLE data [4] from that day. The selected range covers the entire RA-Dec range observed that day.

**Fig. 24:**
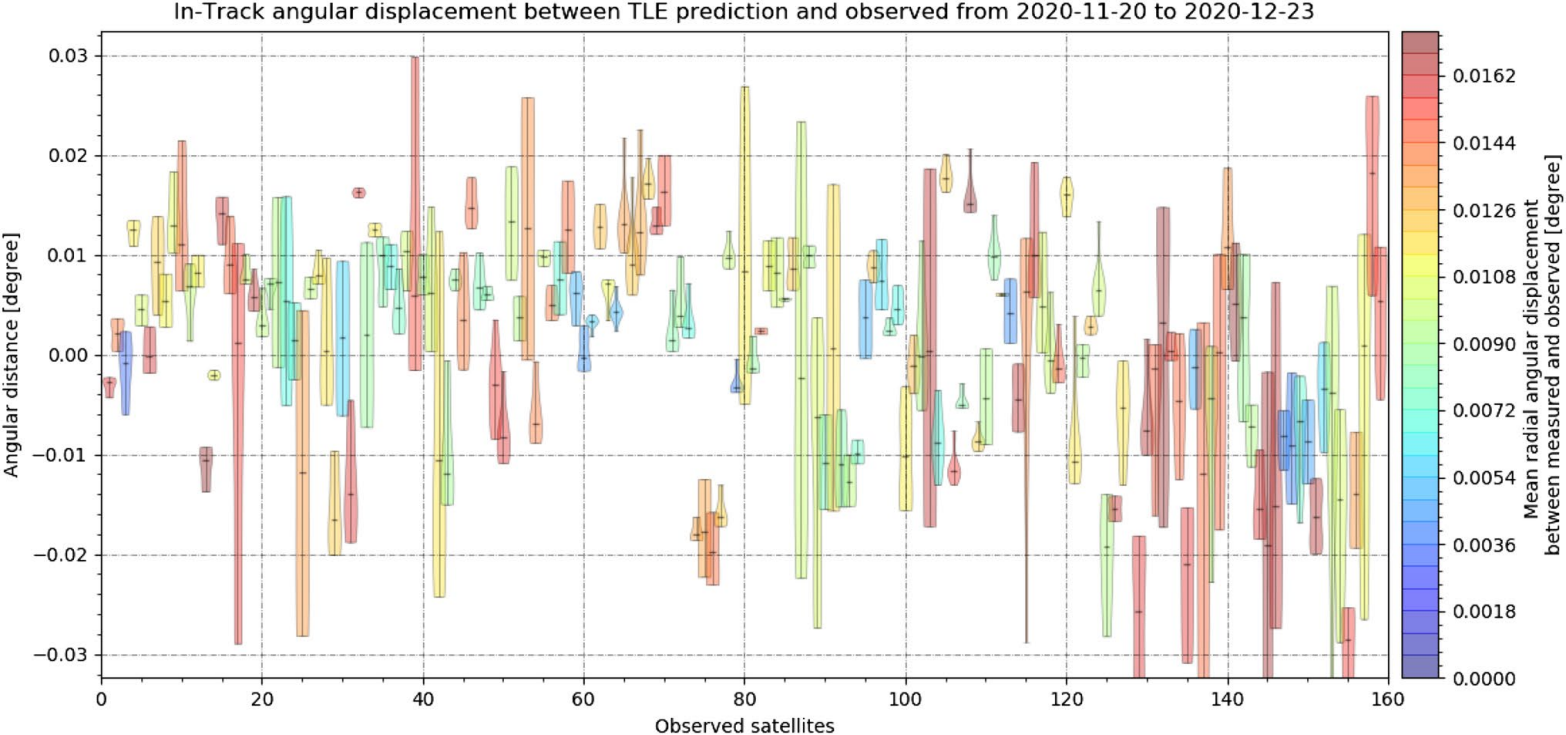
In-track angular displacement between measured object positions and their TLE predictions as a distribution for each object. The color corresponds to the mean angular distance of each object to the TLE prediction (not just in-track) from small values colored in blue to large values colored in red

**Fig. 25:**
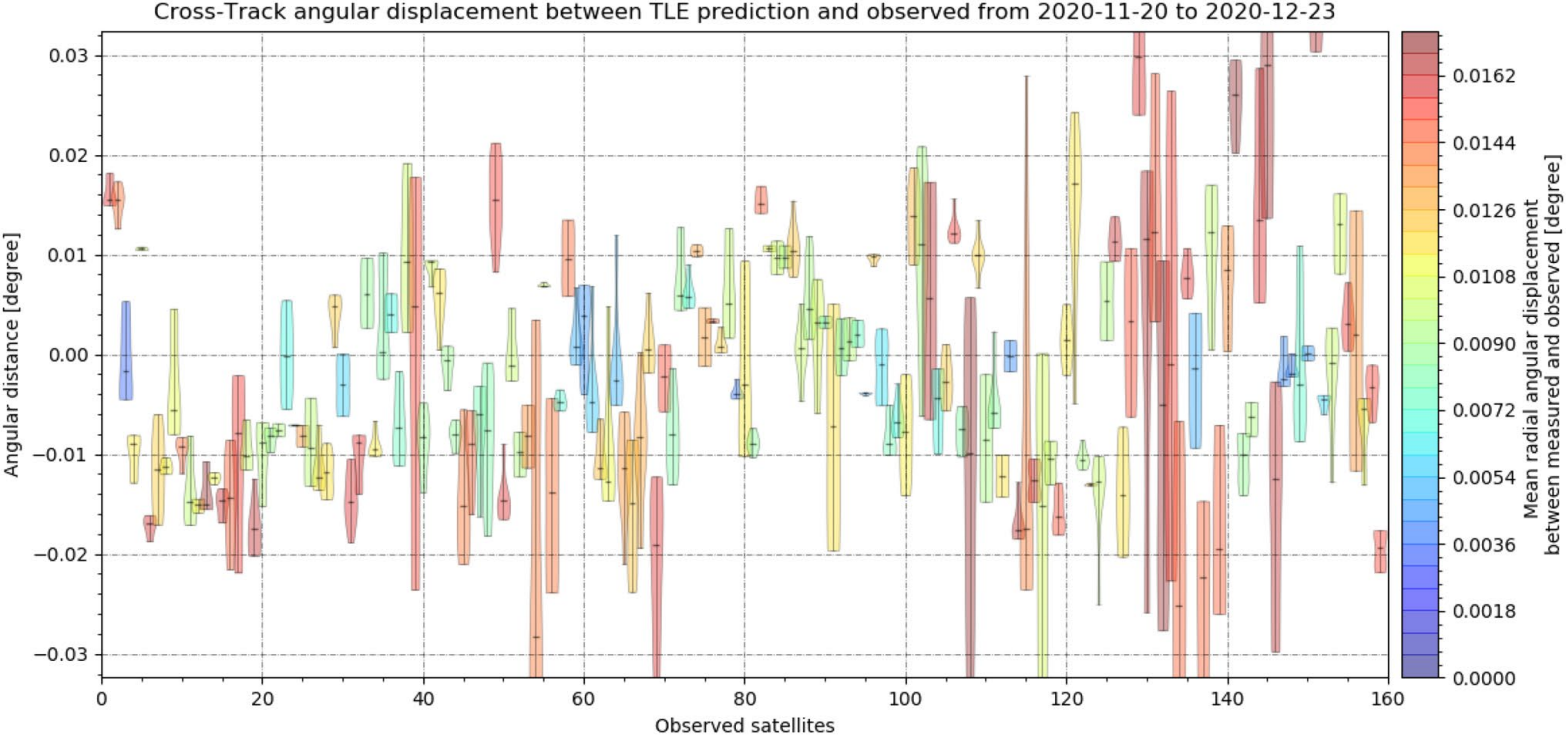
Cross-track angular displacement between measured object positions and their TLE predictions as a distribution for each object. The color corresponds to the mean angular displacement of each object to the TLE prediction (not just cross-track), from small values colored in blue to large values colored in red

The displacement between measurement and predictions shows a constant offset for every object measured. The amount and direction of the angular offset vary between objects. To analyze the angular displacements, the angular displacement is separated into the in-track and cross-track angular displacement. Following Figs. 24, 25 show a subset of the in- and cross-track angular displacement distributions between measurement and TLE prediction, respectively, for single objects.

It can be seen that each single distribution is offset and the offset is mostly larger than the deviation of the angular measurements of a single object. In general, the displacement offsets from all objects scatter around zero. For most measurements, the displacement is equally distributed, but other shapes are visible. Both in- and cross-track errors (offset and deviation) are in general equally large for each object. The median angular deviation from all measured objects is just 0.0041°, but the angular deviation varied from − 0.07° up to 0.05°. The constant offset which was observed between measurements and TLE predictions ranged up to about 0.1°.

**Fig. 26:**
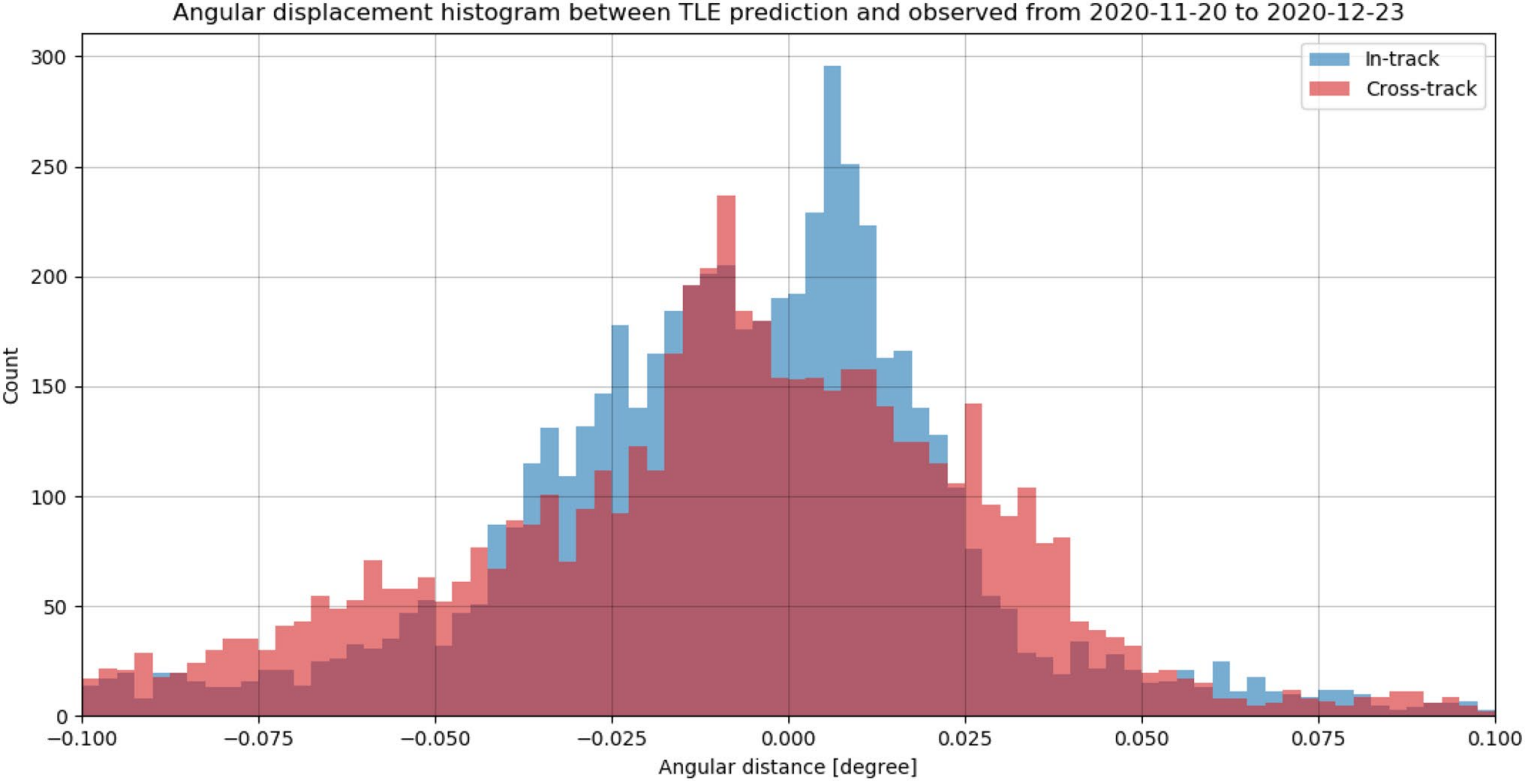
Histogram of angular in- and cross-track displacement between measured object positions and their TLE predictions for all measured and correlated RSO between 20th of November and 23rd of December 2020

Figure 26 shows the histograms of both in- and cross-track angular displacement for all measurements taken by APPARILLO during the December campaign. It can be seen that both displacements scatter around 0, which indicates that there is no systematic error causing the uncertainties. The expected residuals from the TLE predictions are larger in-track than cross-track (0.049° vs. 0.013°) [21]. But the observed displacements show very similar distributions, which is why the observed angular cross-track displacement is a measurement error by the system.

**Fig. 27:**
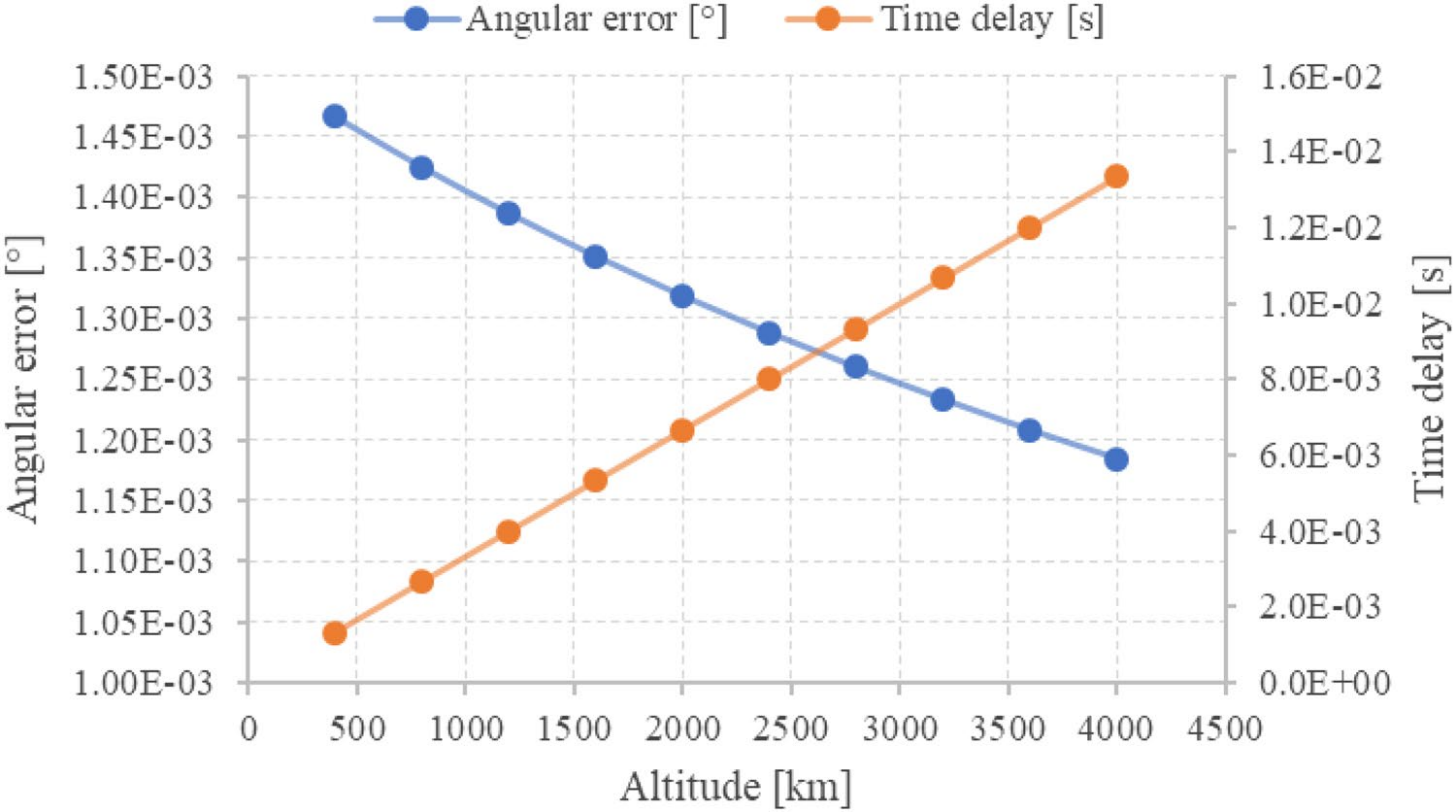
Time delay (orange) of the light received from an RSO and corresponding angular error (blue) depending on its altitude above ground. Circular orbits are assumed to calculate the time delay and angular error

Due to the travel time of the light and the velocity of the RSO, an angular aberration is present. By the time the exposure time is synchronized, the object has already moved and Fig. 27 shows the time delay and corresponding angular error for our system depending on orbit height (assuming a circular orbit). For the observed objects, the angular aberration by RSO’s velocity ranges from 0.0012 to 0.0015°, and is not causing the observed displacement offsets of the distributions. Additionally, no systematic error radial from the image center could be observed, which shows that that lens distortion is not causing the angular error.

Due to large uncertainties of the TLE prediction which ranging from 0.01° for small inclinations in LEO up to 0.05° for large inclination in LEO [21], these results do not give the resulting angular in- and cross-track precision. The mean angular standard deviation of the angular measurements of an object was 0.0041° and showed that streak positions are measured with sub-pixel accuracy. Unfortunately, the mean angular displacement to the prediction is rather large with about 0.05°, and can be considered as first approximation of the angular precision. This is about 7 × larger than the pixel scale of the system (0.007°).

**Fig. 28:**
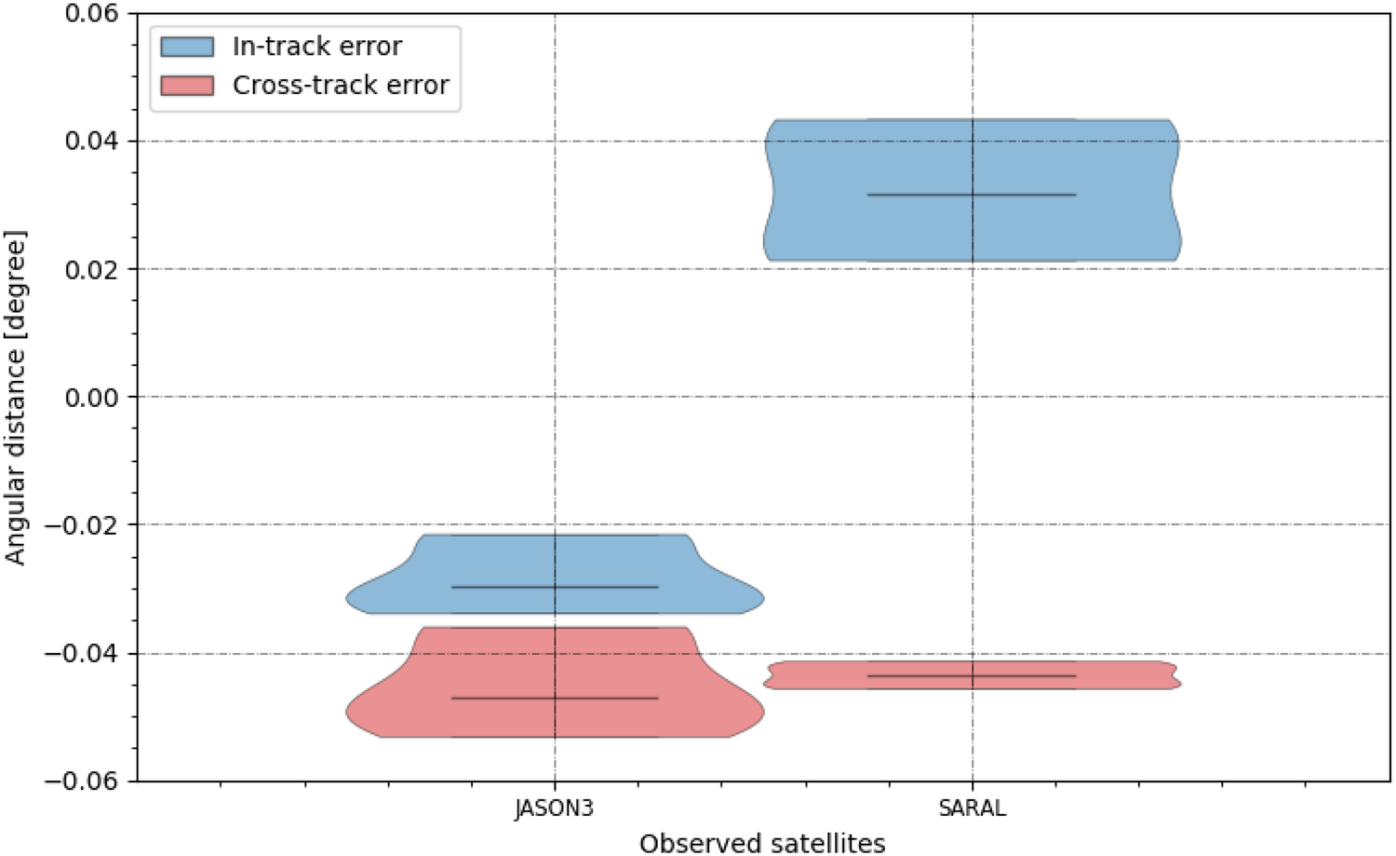
In-track (blue) and cross-track (red) angular displacement distribution between measured object positions and the predicted position using CPF prediction files [24]. The first object is Jason-3 (NORAD = 41240) detected on December 18th and the second SARAL (NORAD = 39086) detected on November 28th

It requires more precise orbit predictions, to determine the absolute precision of the system. Unfortunately, only two of the detected objects had more precise CPF [24] predictions available. For those two, the in- and cross-track angular displacement distributions are shown in Fig. 28.

Both objects show a similar mean angular displacement as observed previously (Figs. 24, 25), which is larger than the deviation of the distribution with 0.02° (Jason-3, NORAD: 41240, COSPAR: 2016-002A) and 0.05° (SARAL, NORAD: 39086, COSPAR: 2013-009A) between measured and predicted positions. The standard deviation of the angular measurement is 0.003° for Jason-3 and 0.006° for SARAL. For the majority of objects, this was observed in the analysis using TLE predictions previously (Figs. 24, 25). These deviations are up to 7 × the systems pixel scale of 0.07°/px (see Table 4) and we can conclude that the estimated system precision is about 0.05° or 7 × the pixel scale. But, these are far too few measurements to conclude the finial precision of the staring system.

## Conclusion

A fully functional staring system was presented which reliably operated between November 20 and December 23, 2020 even under harsh winter weather conditions. The system operated 24/7 during this observation campaign and no false-positive detections were evaluated, which was the major design target of the newly developed image processing. Under optimal observation conditions, the system detected 823 LEO objects within this period with a detection rate up to 36 objects per hour. Multiple detections within one frame can be handled without any constrains. About 15% of observed objects could not be identified using the TLE database [4]. This demonstrates that the presented system is capable of detecting unknown objects and can effectively support subsequent handover to existing database or processing pipelines in form of the TDM format. The detection performance was evaluated using available SATCAT’s RCS and DISCOS data of identified objects. In Figs. 15, 16, it was shown that objects smaller than 1 m^2^ RCS can already be detected, which is very good performance for such a small system. However, the amount of detections smaller than 0.8 m^2^ RCS remains below 10%, see Fig. 17. The smallest objects detected were as small as 0.2 m^2^ in OCS, or 0.25 m^2^ in RCS (see Fig. 21), or 0.045 m^3^ in Volume (see Fig. 18). This is about 2 × larger than theoretical predictions (see Table 1).

The mean angular deviation of a measured object was 0.0041° with a mean angular distance of about 0.05° to the predicted positions. Comparison with precise CPF predictions confirmed this numbers and confirmed that the precision of the system can be estimated as 0.05° or 7 × the pixel scale.

Angular data quality will further improve with improved time synchronization of the image exposure times and velocity aberration correction. Improvements in the image evaluation algorithms should be able to suppress the majority of the false-negative detections to approach near unity detection efficiency, by implementing more sophisticated image processing algorithms like presented by Vananti et al. [25]. A different weather station is recommended to eliminate false clear sky identifications and short image recording during cloudy conditions.

The modular and COTS approach allows simple reproduction of this sensor which is easy to operate within any space surveillance network. This allows to easily adopt the system on future or costumer needs and extend by latest developments like processing techniques. Synthetic tracking is worth to mention as it already showed promising results [26] and is close to real-time processing time with latest consumer GPUs. This will allow an order of magnitude higher sensitivity using the same optical components. This system is the base for future developments to extend space surveillance networks with a small low-cost sensor. Stare and chase with subsequent laser ranging allows immediate cataloging capabilities and was already demonstrated [27].

## Footnotes

1 Raymetrics S.A., 32 Spartis Str, Metamorfosis, GR-14452, Athens, Greece, www.raymetrics.com.

2 Alte Poststraße 24, 88,690 Uhldingen-Mühlhofen, Germany, www.kormoran-tech.com.
